# The Correlation Production in Thermodynamics

**DOI:** 10.3390/e21020111

**Published:** 2019-01-24

**Authors:** Sheng-Wen Li

**Affiliations:** 1 Center for Quantum Technology Research, School of Physics, Beijing Institute of Technology, Beijing 100081, China; lishengwen@bit.edu.cn; 2 Institute for Quantum Science and Engineering, Texas A&M University, College Station, TX 77843, USA

**Keywords:** thermodynamics, macroscopic irreversibility, microscopic reversibility, entropy production, mutual information, correlation production

## Abstract

Macroscopic many-body systems always exhibit irreversible behaviors. However, in principle, the underlying microscopic dynamics of the many-body system, either the (quantum) von Neumann or (classical) Liouville equation, guarantees that the entropy of an isolated system does not change with time, which is quite confusing compared with the macroscopic irreversibility. We notice that indeed the macroscopic entropy increase in standard thermodynamics is associated with the correlation production inside the full ensemble state of the whole system. In open systems, the irreversible entropy production of the open system can be proved to be equivalent with the correlation production between the open system and its environment. During the free diffusion of an isolated ideal gas, the correlation between the spatial and momentum distributions is increasing monotonically, and it could well reproduce the entropy increase result in standard thermodynamics. In the presence of particle collisions, the single-particle distribution always approaches the Maxwell-Boltzmann distribution as its steady state, and its entropy increase indeed indicates the correlation production between the particles. In all these examples, the total entropy of the whole isolated system keeps constant, while the correlation production reproduces the irreversible entropy increase in the standard macroscopic thermodynamics. In this sense, the macroscopic irreversibility and the microscopic reversibility no longer contradict with each other.

## 1. Introduction

**Figure 1:**
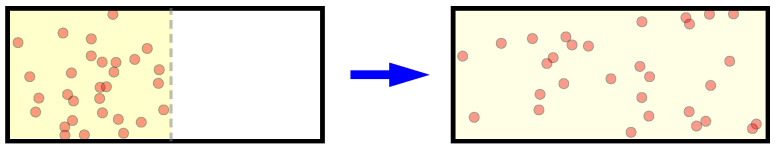
Demonstration for gas diffusion.

Considering an isolated ideal gas with *N* particles initially occupying only part of a box, after long enough time diffusion, the gas spreads all over the volume uniformly (Figure 1). From the standard macroscopic thermodynamics it is simple to show the gas entropy is increased by $\Delta S=Nk_{B}\ln\left(V/V_{0}\right)$, where *V* ($V_{0}$) is the final (initial) occupied volume [1,2].

This is a quite typical example of the entropy increase in the macroscopic thermodynamics. However, notice that isolated quantum systems always follow the unitary evolution, and the system density matrix $\hat{\rho }\left(t\right)$ is described by the von Neumann equation $\partial_{t}\hat{\rho }=i\left[\hat{\rho },\hat{H}\right]$, which guarantees the von Neumann entropy $S_{V}\left[\hat{\rho }\right]=-\mathrm{tr}\left[\hat{\rho }\ln\hat{\rho }\right]$ does not change with time. In principle, this result should also apply for many-body systems, then it seems inconsistent with the above entropy increase in the standard macroscopic thermodynamics.

Indeed, this is not a problem that only appears in quantum physics, and classical physics has the same situation. For an isolated classical system, the ensemble evolution follows the Liouville equation [1,3,4],

(1) $$\partial_{t}\rho \left(\vec{P},\vec{Q},t\right)=-\left\{\rho \left(\vec{P},\vec{Q},t\right),\;H\right\},$$

which is derived from the Hamiltonian dynamics. Here {·,·} is the Poisson bracket, and $\rho (\vec{P},\vec{Q},t)$ is the probability density around the microstate $\left(\vec{P},\vec{Q}\right):=\left({\vec{p}}_{1},{\vec{p}}_{2},\ldots ;\;{\vec{q}}_{1},{\vec{q}}_{2},\ldots \right)$ at time *t*. As a result, the Gibbs entropy of the whole system keeps a constant and never changes with time,

(2) $$\frac{d}{dt}S_{G}\left[\rho \left(\vec{P},\vec{Q},t\right)\right]=\frac{d}{dt}\left[-\int d^{3N}p\;d^{3N}q\;\rho \ln\rho \right]=0.$$

Therefore, this constant entropy result exists in both quantum and classical physics.

This is rather confusing when compared with our intuition of the “irreversibility” happening in the macroscopic world [4,5,6,7,8,9,10,11]. Moreover, even if the particles have complicated nonlinear interactions, although the system dynamics could be highly chaotic and unpredictable, the (classical) Liouville or (quantum) unitary dynamics still guarantees the entropy of isolated systems does not change with time.

Here we need to make some clarification on the word “irreversibility”. In thermodynamics, a “reversible (irreversible)” process means the system is (not) always in the thermal equilibrium state at every moment. Throughout this paper, we adopt the meaning in dynamics: for any initial condition, if some function (distribution, state, etc.) always approaches the same steady state, then such kind of behavior is regarded as “irreversible”.

On the other hand, if there is no inter-particle interaction, the microstate evolution is well predictable, but the above macroscopic diffusion process still happens irreversibly until the gas achieves the new uniform distribution in the whole volume. From this sense, it seems that the above contradiction between the constant entropy and the appearance of macroscopic irreversibility does not depend on whether there exist complicated interactions. Thus we need to ask: how could the macroscopic irreversibility and entropy increase arise from the underlying microscopic dynamics, which is reversible with time-reversal symmetry [6,12]?

Recently, it is noticed that the irreversible entropy production in open systems indeed is deeply related with correlation between the open system and its environment [13,14,15,16]. In an open system, the entropy of the system itself can either increase or decrease, depending on whether it is absorbing or emitting heat to its environment. Subtracting such thermal entropy due to the heat exchange, the rest part of the system entropy change is called the irreversible entropy production [17,18,19,20,21], and that increases monotonically with time until reaching the thermal equilibrium.

Under proper approximations, we can prove indeed the thermal entropy change due to the heat exchange is just equal to the entropy change of the environment state [16,22,23], and the irreversible entropy production is equivalent as the correlation generation between the open system and its environment, which is measured by the relative entropy [13,14,24,25,26] or their mutual information [16,23]. At the same time, the system and its environment together as a whole system maintains constant entropy during the evolution. In this sense, the constant global entropy and the increase of the system-environment correlation well coincide with each other. Moreover, when the baths are non-thermal states, which are beyond the application scope of the standard macroscopic thermodynamics, we could see such correlation production still applies (see Section 2.4).

That is to say, due to the practical restrictions of measurements, indeed some correlation information hiding in the global state is difficult to be sensed, and that results to the appearance of the macroscopic irreversibility as well as the entropy increase. In principle, such correlation understanding could also apply for isolated systems. Indeed, in the above diffusion example, our observation that “the gas spread all over the volume uniformly” is implicitly focused on the spatial distribution only, rather than the total ensemble state.

For the classical ideal gas with no inter-particle interactions, the Liouville equation for the ensemble evolution can be exactly solved [4,9,27]. Notice that in practice, it is the spatial and momentum distributions that are directly measured, but not the full ensemble state. We can prove that the spatial distribution $P_{x}\left(x,t\right)$, as a marginal distribution of the whole ensemble, always approaches the new uniform one as its steady state. Moreover, by examining the correlation between the spatial and momentum distributions, we can see their correlation increases monotonically, and could reproduce the entropy increase in standard thermodynamics. At the same time, the total ensemble state $\rho (\vec{P},\vec{Q},t)$ keeps constant entropy during the diffusion process (see Section 3).

For the non-ideal gas with weak particle interactions, the dynamics of the single-particle probability distribution function (PDF) f(p,r,t) can be described by the Boltzmann equation [1,8]. According to the Boltzmann *H*-theorem, f(p,r,t) always approaches the Maxwell-Boltzmann (MB) distribution as its steady state, and its entropy increases monotonically. Notice that the single-particle PDF f(p,r,t) is a marginal distribution of the full ensemble state $\rho (\vec{P},\vec{Q},t)$, which is obtained by averaging out all the other particles. Thus f(p,r,t) does not contain the particle correlations, and the increase of its entropy indeed implicitly reflects the increase of the inter-particle correlations, which exactly reproduces the entropy increase result in the standard macroscopic thermodynamics. At the same time, the total ensemble $\rho (\vec{P},\vec{Q},t)$ still follows the Liouville equation with constant entropy.

The correlation production between the particles could also help us understand the Loschmidt paradox: when we consider the “backward” evolution, since significant particle correlations have established [28], the molecular-disorder assumption, which is the most crucial approximation in deriving the Boltzmann equation, indeed does not hold. Therefore, the Boltzmann equation as well as the *H*-theorem of entropy increase does not apply for the “backward” evolution (see Section 4).

In sum, the global state keeps constant entropy, but in practice, usually it is the partial information (e.g., marginal distribution, single-particle observable expectations) that is directly accessible to our observation, and that gives rise to the appearance of the macroscopic irreversibility [9,13,14,15,16,23,24,25,27,29,30,31,32,33,34]. The entropy increase in the standard macroscopic thermodynamics indeed reflects the correlation increase between different degrees of freedom (DoF) in the many-body system. In this sense, the reversibility of microscopic dynamics (for the global state) and the macroscopic irreversibility (for the partial information) coincide with each other. More importantly, this correlation understanding applies for both quantum and classical systems, and for both open and isolated systems; besides, it does not depends on whether there exist complicated particle interactions, and also can be used to describe time-dependent non-equilibrium systems.

## 2. The Correlation Production in Open Systems

In this section, we first discuss the thermodynamics of an open system, which is surrounded by an environment exchanging energy with it. The open system can absorb or emit heat to the environment, as a result, the entropy of the open system itself can either increase or decrease. Thus the thermodynamic irreversibility is not simply related to the entropy change of the open system alone, but should be described by the “irreversible entropy”, which increases monotonically with time.

Here we first give a brief review about this formalism for the irreversible entropy production, which is an equivalent statement for the second law. Then we will show indeed this irreversible entropy production in open systems is just equivalent with the correlation increase between the system and its environment [13,16,29], which is measured by their mutual information. Moreover, if the baths contacting with the system are not canonical thermal ones, the temperatures are no longer well defined, and this situation is indeed not within the applicable scope of the second law in standard thermodynamics, but we will see the the correlation production still applies in this case.

### 2.1. The Irreversible Entropy Production Rate

Now we first briefly review the formalism of entropy production [17,18,19,20,21]. The entropy change dS of an open system can be regarded as coming from two origins, i.e.,

(3) $$dS=dS_{e}+dS_{i}$$

where $dS_{e}$ comes from the heat exchange with external baths, and $dS_{i}$ is regarded as the irreversible entropy change. The exchanging part $dS_{e}$ can be either positive or negative, indicating the heat absorbing or emitting of the system. But the irreversible entropy change $dS_{i}$, as stated by the second law, should always be positive.

If the system is contacted with a thermal bath in the equilibrium state with temperature *T*, the entropy change due to the heat exchange can be written as $dS_{e}=đQ/T$ (hereafter we refer it as the thermal entropy), where đQ is the heat absorbed by the system. Then the second law can be expressed as

(4) $$dS_{i}=dS-\frac{đQ}{T}\geq 0,$$

where the equality holds only for reversible processes. This is just the Clausius inequality for an infinitesimal process [1,18,21].

**Figure 2:**
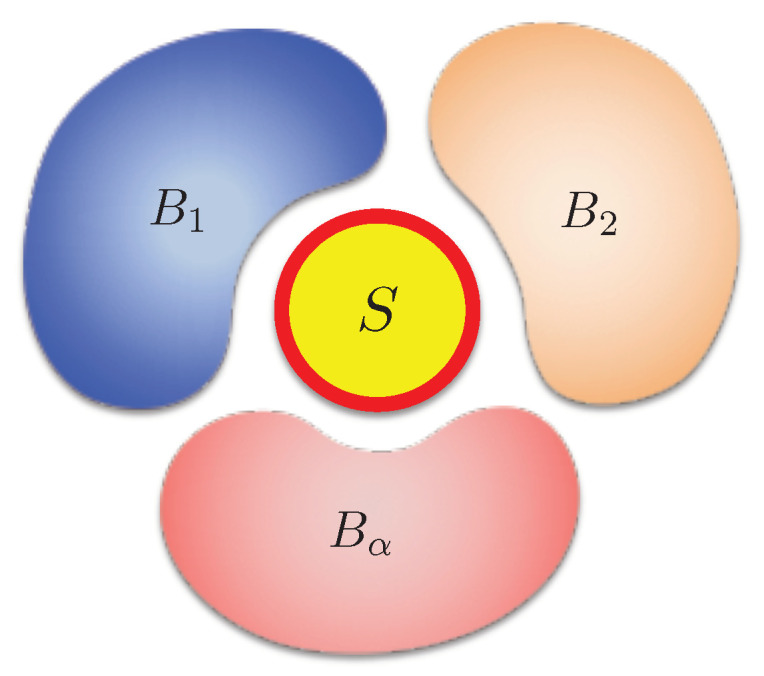
Demonstration for an open system *S* surrounded by several independent baths $B_{\alpha }$.

More generally, if the system contacts with multiple independent thermal baths with different temperatures $T_{\alpha }$ at the same time (Figure 2), the irreversible entropy change should be generalized as [17]

(5) $$dS_{i}=dS-\sum_{\alpha }\frac{đQ_{\alpha }}{T_{\alpha }}\geq 0,$$

where $đQ_{\alpha }$ is the heat absorbed from bath-α [18,21]. For example, for a system contacting with two thermal baths with temperatures $T_{1,2}$, in the steady state, we have dS=0 and $đQ_{1}=-đQ_{2}$, thus the above equation gives [21]

(6) $$-đQ_{1}\left(\frac{1}{T_{1}}-\frac{1}{T_{2}}\right)\geq 0.$$

It is easy to verify $đQ_{1}=-đQ_{2}>0$ always comes together with $T_{1}>T_{2}$, and vice versa. That means, the heat always flows from the high temperature area to the low temperature area, which is just the Clausius statement of the second law.

Therefore, for an open system, the second law can be equivalently expressed as a simple inequality $dS_{i}\geq 0$, which means the irreversible entropy change always increases monotonically. This can be also expressed by the entropy production rate (EPr), which is defined as

(7) $$R_{\mathrm{EP}}:=\frac{dS_{i}}{dt}=\frac{dS}{dt}-\sum_{\alpha }\frac{1}{T_{\alpha }}\frac{dQ_{\alpha }}{dt},$$

and $R_{\mathrm{EP}}\geq 0$ is equivalent as saying the irreversible entropy keeps increasing.

Besides the equivalence with the standard second law statements, the entropy production formalism also provides a proper way to quantitively study the non-equilibrium thermodynamics. Considering there is only one thermal bath, the system would get thermal equilibrium with the bath in the steady state. At this time, the system state no longer changes, and there is no net heat exchange between the system and the bath, thus $R_{\mathrm{EP}}\to 0$ when t→∞.

In contrast, if the system contacts with multiple thermal baths with different temperatures, in the steady state, although the system state no longer changes with time, there still exists net heat flux between the system and the baths. Therefore, different from the thermal equilibrium, such a steady state is a stationary non-equilibrium state [17]. Notice that in this case the EPr remains a finite positive value $R_{\mathrm{EP}}>0$ (see the example of Equation (6)) when t→∞, which indicates there is still on-going production of irreversible entropy. Therefore, $R_{\mathrm{EP}}=0$ (or >0) well indicates whether (or not) the system is in the thermal equilibrium state.

Here the above discussions about the entropy production apply for both classical and quantum systems, as long as the quantities like $\dot{S}$ and ${\dot{Q}}_{\alpha }$ are calculated by the classical ensemble or quantum state correspondingly.

### 2.2. The Production Rate of the System-Bath Correlation

Now we will show the above EPr is indeed equivalent as the production rate of the correlation between the open system and its environment. Usually the dynamics of the open system alone is more often concerned in literature. The baths, due to their large size, are usually considered as unaffected by the system, and only provides a background with fluctuations. But the system surely has influence to its environment [16,23,35]. For example, when the system emits energy, this energy is indeed added to the environment. To study the correlation between the system and its environment, here we also need to know the dynamics of the whole environment.

#### 2.2.1. Quantum Case

Here we first consider a quantum system contacting with several independent thermal baths with temperatures $T_{\alpha }$. Initially, each bath-α stays in the canonical thermal state

(8) $${\hat{\rho }}_{B,\alpha }\left(0\right)=\frac{1}{Z_{\alpha }}\exp\left[-\frac{{\hat{H}}_{B,\alpha }}{T_{\alpha }}\right],$$

with $Z_{\alpha }$ as the normalization factor. The exact changing rate of the information entropy of bath-α is given by $\frac{d}{dt}S_{B,\alpha }\left(t\right)=-\mathrm{tr}\left[{\dot{\hat{\rho }}}_{B,\alpha }\left(t\right)\ln{\hat{\rho }}_{B,\alpha }\left(t\right)\right]$. To make further calculation, we assume the bath state ${\hat{\rho }}_{B,\alpha }\left(t\right)$ does not change too much from the initial state, thus $\ln{\hat{\rho }}_{B,\alpha }\left(t\right)=\ln\left[{\hat{\rho }}_{B,\alpha }\left(0\right)+\delta {\hat{\rho }}_{t}\right]≃\ln{\hat{\rho }}_{B,\alpha }\left(0\right)+o\left(\delta {\hat{\rho }}_{t}\right)$, then the bath entropy change ${\dot{S}}_{B,\alpha }\left(t\right)$ becomes [16,22,23,26]

(9) $$\begin{array}{cc} {\dot{S}}_{B,\alpha }\left(t\right) & ≃-\mathrm{tr}[{\dot{\hat{\rho }}}_{B,\alpha }\left(t\right)\ln{\hat{\rho }}_{B,\alpha }\left(0\right)]\\ & =-\mathrm{tr}\left\{{\dot{\hat{\rho }}}_{B,\alpha }\left(t\right)\cdot \ln\left(\frac{1}{Z_{\alpha }}\exp\left[-\frac{{\hat{H}}_{B,\alpha }}{T_{\alpha }}\right]\right)\right\}=\frac{1}{T_{\alpha }}\frac{d}{dt}\langle {\hat{H}}_{B,\alpha }\rangle . \end{array}$$

Notice that here $\frac{d}{dt}\langle {\hat{H}}_{B,\alpha }\rangle$ is the energy increase of bath-α, thus it is just equal to the energy loss of the system to bath-α (i.e., $-{\dot{Q}}_{\alpha }$) when the system-bath interaction strength is negligibly small. Therefore, the above EPr $R_{\mathrm{EP}}$ (Equation (7)) can be rewritten as $R_{\mathrm{EP}}≃{\dot{S}}_{S}\left(t\right)+\sum_{\alpha }{\dot{S}}_{B,\alpha }\left(t\right)$.

Since initially the different baths are independent from each other and do not interact with each other directly, we assume they cannot generate significant correlations during the evolution, thus the entropy of the whole environment is simply the summation of that from each single bath, namely, $S_{B}\left(t\right)≃\sum_{\alpha }S_{B,\alpha }\left(t\right)$. Therefore, the above EPr $R_{\mathrm{EP}}$ can be further rewritten as

(10) $$R_{\mathrm{EP}}≃{\dot{S}}_{S}\left(t\right)+{\dot{S}}_{B}\left(t\right)=\frac{d}{dt}\left[S_{S}+S_{B}-S_{\mathrm{SB}}\right]=\frac{d}{dt}I_{\mathrm{SB}}\left(t\right).$$

Here $S_{\mathrm{SB}}$ is the von Neumann entropy of the whole s + b state, which does not change with time ($\frac{d}{dt}S_{\mathrm{SB}}=0$), since the whole s + b system is an isolated system and follows the unitary evolution [36].

Therefore, the production rate of the irreversible entropy $R_{\mathrm{EP}}$ is just equivalent with the production rate of the mutual information between the system and its environment, $I_{\mathrm{SB}}=S_{S}+S_{B}-S_{\mathrm{SB}}$, which measures their correlation [36]. That means, the second law statement that the irreversible entropy keeps increasing ($R_{\mathrm{EP}}\geq 0$) can be also equivalently understood as, the correlation between the system and its environment, as measured by the mutual information, always keeps increasing until they get the equilibrium.

#### 2.2.2. Classical Case

The above discussions about quantum open systems also applies for classical ones. For classical systems, the initial state of bath-α should be represented by the canonical ensemble distribution

(11) $$\rho_{B,\alpha }\left(\vec{P},\vec{Q},t=0\right)=\frac{1}{Z_{\alpha }}\exp\left[-\frac{1}{T_{\alpha }}H_{B,\alpha }\left(\vec{P},\vec{Q}\right)\right],$$

where $\left(\vec{P},\vec{Q}\right):=\left({\vec{p}}_{1},{\vec{p}}_{2},\ldots ;{\vec{q}}_{1},{\vec{q}}_{2},\ldots \right)$ denotes the momenta and positions of the DoF in bath-α. Then we consider the changing rate of the Gibbs entropy of bath-α, and that is

(12) $$\begin{array}{cc} \frac{d}{dt}S_{G}\left[\rho_{B,\alpha }\left(\vec{P},\vec{Q},t\right)\right] & =-\int d^{3N}p\;d^{3N}q\;\partial_{t}\rho_{B,\alpha }\left(t\right)\ln\rho_{B,\alpha }\left(t\right)≃-\int d^{3N}p\;d^{3N}q\;\partial_{t}\rho_{B,\alpha }\left(t\right)\ln\rho_{B,\alpha }\left(0\right)\\ & =-\int d^{3N}p\;d^{3N}q\;\partial_{t}\rho_{B,\alpha }\left(t\right)\cdot \left[-\frac{1}{T_{\alpha }}H_{B,\alpha }\left(\vec{P},\vec{Q}\right)\right]=\frac{1}{T_{\alpha }}\frac{d}{dt}\langle H_{B,\alpha }\left(\vec{P},\vec{Q}\right)\rangle . \end{array}$$

Here we adopted the similar approximation $\ln\rho_{B,\alpha }\left(\vec{P},\vec{Q},t\right)≃\ln\rho_{B,\alpha }\left(\vec{P},\vec{Q},0\right)$ as above, and this result is simply the classical counterpart of Equation (9).

Therefore, for classical open systems, the EPr $R_{\mathrm{EP}}$ in Equation (7) also can be rewritten as $R_{\mathrm{EP}}=\frac{d}{dt}\left(S_{S}+S_{B}\right)$. Furthermore, since the whole s + b system is an isolated system, its dynamics follows the Liouville equation, thus the Gibbs entropy of the whole s + b system does not change with time, i.e., $\frac{d}{dt}S_{\mathrm{SB}}=0$. Therefore, the equivalence between the irreversible entropy production and the system-bath correlation (Equation (10)) also holds for classical systems. That means, for both classical and quantum open systems contacting with thermal baths, the second law can be equivalently stated as, the correlation between the system and its environment, which is measured by their mutual information, always keeps increasing.

### 2.3. Master Equation Representation

Besides the above general discussions, the time-dependent dynamics of the open system, either classical or quantum, can be quantitively described by a master equation. With the help of the master equations, the above EPr can be further written in a more detailed form. Here we will show this for both classical and quantum cases.

#### 2.3.1. Classical Case

For a classical open system, the interaction with the baths would lead to the probability transition between its different states, and this dynamics is usually described by the Pauli master equation [37]

(13) $${\dot{p}}_{n}=\sum_{\alpha }\sum_{m}L_{n\leftarrow m}^{\left(\alpha \right)}\;p_{m}-L_{m\leftarrow n}^{\left(\alpha \right)}\;p_{n},$$

which is a Markovian process. Here $p_{n}$ is the probability to find the system in state-*n* (whose energy is $E_{n}$), and $L_{n\leftarrow m}^{\left(\alpha \right)}$ is the probability transition rate from state-*m* to state-*n* due to the interaction with the thermal bath-α. The back and forth transition rates between states-m,n should satisfy the following ratio [17,38]

(14) $$\frac{L_{m\leftarrow n}^{\left(\alpha \right)}}{L_{n\leftarrow m}^{\left(\alpha \right)}}=\exp\left[-\frac{1}{T_{\alpha }}\left(E_{m}-E_{n}\right)\right],$$

which means the “downward” transition to the low energy state is faster than the “upward” one by a Boltzmann factor. In the case of only one thermal bath, with this relation, the detailed balance $L_{n\leftarrow m}^{\left(\alpha \right)}\;p_{m}-L_{m\leftarrow n}^{\left(\alpha \right)}\;p_{n}=0$ simply leads to the Boltzmann distribution $p_{n}:p_{m}=e^{-E_{n}/T}:e^{-E_{m}/T}$ in the steady state.

If there are multiple thermal baths, the energy average $\langle E\rangle =\sum_{n}E_{n}p_{n}$ gives an energy-flow conservation relation

(15) $$\partial_{t}\langle E\rangle =\sum_{\alpha }J_{\alpha },\;J_{\alpha }:=\sum_{m,n}\left(L_{n\leftarrow m}^{\left(\alpha \right)}\;p_{m}-L_{m\leftarrow n}^{\left(\alpha \right)}\;p_{n}\right)E_{n},$$

thus $J_{\alpha }$ is the heat current flowing into the system from bath-α (${\dot{Q}}_{\alpha }$). We can put these relations, as well as the Gibbs entropy of the system $S_{G}=-\sum_{n}p_{n}\ln p_{n}$, into the above EPr (7), obtaining [39]

(16) $$\begin{array}{cc} R_{\mathrm{EP}} & =\sum_{\alpha }\sum_{m,n}-\left(L_{n\leftarrow m}^{\left(\alpha \right)}\;p_{m}-L_{m\leftarrow n}^{\left(\alpha \right)}\;p_{n}\right)\ln p_{n}-\frac{E_{n}}{T_{\alpha }}\left(L_{n\leftarrow m}^{\left(\alpha \right)}\;p_{m}-L_{m\leftarrow n}^{\left(\alpha \right)}\;p_{n}\right)\\ & =\sum_{\alpha }\sum_{m,n}\frac{1}{2}\left(L_{n\leftarrow m}^{\left(\alpha \right)}\;p_{m}-L_{m\leftarrow n}^{\left(\alpha \right)}\;p_{n}\right)\left(\ln\frac{e^{-E_{n}/T_{\alpha }}}{p_{n}}-\ln\frac{e^{-E_{m}/T_{\alpha }}}{p_{m}}\right)\\ & =\sum_{\alpha }\sum_{m,n}\frac{1}{2}\left(L_{n\leftarrow m}^{\left(\alpha \right)}\;p_{m}-L_{m\leftarrow n}^{\left(\alpha \right)}\;p_{n}\right)\;\ln\left(L_{n\leftarrow m}^{\left(\alpha \right)}\;p_{m}/L_{m\leftarrow n}^{\left(\alpha \right)}\;p_{n}\right). \end{array}$$

Notice that each summation term must be non-negative, thus we always have $R_{\mathrm{EP}}\geq 0$, which is just consistent with the above second law statement that the irreversible entropy keeps increasing. $R_{\mathrm{EP}}=0$ holds only when $L_{n\leftarrow m}^{\left(\alpha \right)}\;p_{m}=L_{m\leftarrow n}^{\left(\alpha \right)}\;p_{n}$ for any α, and this is possible only when all the baths have the same temperature, which means the thermal equilibrium. Otherwise, in the steady state, although it is time-independent, there still exists non-equilibrium flux flowing across the system, and that is indicated by $R_{\mathrm{EP}}>0$.

#### 2.3.2. Quantum Case

For a quantum system weakly coupled with the multiple thermal baths, usually its dynamics can be described by the GKSL (Lindblad) equation [40,41],

(17) $$\dot{\hat{\rho }}=i\left[\hat{\rho },{\hat{H}}_{S}\right]+\sum_{\alpha }L_{\alpha }\left[\hat{\rho }\right].$$

where $\hat{\rho }$ is the system state and $L_{\alpha }\left[\hat{\rho }\right]$ describes the dissipation due to bath-α. Using the von Neumann entropy $S_{V}\left[\hat{\rho }\right]=-\mathrm{tr}\left[\hat{\rho }\ln\hat{\rho }\right]$ and heat current ${\dot{Q}}_{\alpha }=\mathrm{tr}\left[{\hat{H}}_{S}\cdot L_{\alpha }\left[\hat{\rho }\right]\right]$, the EPr (7) can be rewritten as the following Spohn formula [39,42,43,44,45,46,47]

(18) $$\begin{array}{cc} R_{\mathrm{EP}} & =-\mathrm{tr}\left[\dot{\hat{\rho }}\ln\hat{\rho }\right]+\sum_{\alpha }\mathrm{tr}\left[L_{\alpha }\left[\hat{\rho }\right]\cdot \ln{\hat{\rho }}_{\mathrm{SS}}^{\left(\alpha \right)}\right]\\ & =\sum_{\alpha }\mathrm{tr}\left[\left(\ln{\hat{\rho }}_{\mathrm{SS}}^{\left(\alpha \right)}-\ln\hat{\rho }\right)L_{\alpha }\left[\hat{\rho }\right]\right]:=R_{\mathrm{Sp}}. \end{array}$$

Here ${\hat{\rho }}_{\mathrm{SS}}^{\left(\alpha \right)}$ satisfies $L_{\alpha }\left[{\hat{\rho }}_{\mathrm{SS}}^{\left(\alpha \right)}\right]=0$, and we call it the *partial steady state* associated with bath-α. If the system only interacts with bath-α, then ${\hat{\rho }}_{\mathrm{SS}}^{\left(\alpha \right)}$ should be its steady state when t→∞. Clearly, ${\hat{\rho }}_{\mathrm{SS}}^{\left(\alpha \right)}$ should be the thermal state ($∼\exp[-{\hat{H}}_{S}/T_{\alpha }]$) when bath-α is the canonical thermal one with temperature $T_{\alpha }$, and the term $\chi_{\alpha }:=\mathrm{tr}\left[L_{\alpha }\left[\hat{\rho }\right]\cdot \ln{\hat{\rho }}_{\mathrm{SS}}^{\left(\alpha \right)}\right]=-{\dot{Q}}_{\alpha }/T_{\alpha }$ is the corresponding exchange of thermal entropy.

The positivity of $R_{\mathrm{Sp}}$ is not so obvious as the classical case (16), but still we can prove $R_{\mathrm{Sp}}\geq 0$, if the master Equation (17) has the standard GKSL form (see the proof in Appendix of Reference [16] or References [42,43]). The GKSL form of the master Equation (17) indicates it describes a Markovian process [40,41,48], which is similar like the above classical case. Again this is consistent with the above discussions about the second law statement.

#### 2.3.3. Remark

In the above discussions, we focused on the case that the baths are canonical thermal ones. As a result, in the above master equations, the transition rate ratios (14) appear as the Boltzmann factors, and the partial steady states ${\hat{\rho }}_{\mathrm{SS}}^{\left(\alpha \right)}$ of the system are the canonical thermal states. Strictly speaking, only for canonical thermal baths, the temperature *T* is well defined, and the thermal entropy $dS_{e}=đQ/T$ can be applied, as well as the above EPr (7), which is the starting point to derive the master equation representations (Equations (16) and (18)).

If the baths are non-thermal states, there is no well-defined temperature, thus the above EPr in standard thermodynamics in Section 2.1, especially the thermal entropy $dS_{e}=dQ/T$, does not apply. But master equations still can be used to study the dynamics of such systems. Due to the interaction with non-thermal baths, the transition rate ratios (14) do not need to be the Boltzmann factors, but we can verify the last line of Equation (16) still remains positive. Thus Equation (16) can be regarded as a generalized EPr beyond standard thermodynamics, however, now it is unclear to tell its physical meaning, as well as its relation with the non-thermal bath.

The quantum case has the same situation. If the master equation (17) comes from non-thermal baths, the partial steady state ${\hat{\rho }}_{\mathrm{SS}}^{\left(\alpha \right)}$ would not be the thermal state with the temperature of bath-α, but the quantity $R_{\mathrm{Sp}}$ (last line of Equation (18)) could still remain positive [16,42,43]. However, in this case the physical meaning of the Spohn formula (18) is not clear now.

In the following example of an open quantum system interacting with non-thermal baths, we will show that, although it is beyond the applicable scope of standard thermodynamics, the Spohn formula (18) is still equal to the production rate of the system-bath correlation, which is the same as the thermal bath case in Section 2.2, and the term $\chi_{\alpha }=\mathrm{tr}\left[L_{\alpha }\left[\hat{\rho }\right]\cdot \ln{\hat{\rho }}_{\mathrm{SS}}^{\left(\alpha \right)}\right]$ is just equal to the informational entropy change of bath-α.

### 2.4. Contacting with Squeezed Thermal Baths

When the heat baths contacting with the system are not canonical thermal ones, it is possible to construct a heat engine that “seemingly” works beyond the Carnot bound. For example, in an optical cavity, a collection of atoms with non-vanishing quantum coherence can be used to generate light force to do mechanical work by pushing the cavity well [49]; a squeezed light field can be used to as the reservoir for an harmonic oscillator which expands and compresses as a heat engine [50]. In these studies, it seems that the efficiency of the heat engine could be higher than the Carnot bound $\eta_{C}=1-T_{c}/T_{h}$. However, since the baths are not canonical thermal ones, the parameter *T* can no longer be regarded as the well defined temperature. As we have emphasized, such kind of systems are indeed not within the applicable scope of standard thermodynamics, therefore they do not need to obey the second law inequalities that are based on canonical thermal baths [51].

In this non-thermal bath case, the thermal entropy $dS_{e}=đQ/T$ does not apply, but the information entropy is still well defined. Now we study the system-bath mutual information when the baths are non-thermal states [16]. We consider an example of a single mode boson (${\hat{H}}_{S}=\Omega {\hat{a}}^{†}\hat{a}$) which is linearly coupled with multiple squeezed thermal baths (${\hat{H}}_{B}=\sum_{\alpha }{\hat{H}}_{B,\alpha }$ and ${\hat{H}}_{B,\alpha }=\sum_{k}\omega_{\alpha k}\;{\hat{b}}_{\alpha k}^{†}{\hat{b}}_{\alpha k}$), and they interact through ${\hat{V}}_{\mathrm{SB}}=\sum_{\alpha }g_{\alpha k}{\hat{a}}^{†}{\hat{b}}_{\alpha k}+g_{\alpha k}^{*}\hat{a}{\hat{b}}_{\alpha k}^{†}$. The initial states of the baths are squeezed thermal ones,

(19) $$\begin{array}{c} {\hat{\rho }}_{B,\alpha }\left(0\right)=\frac{1}{Z_{\alpha }}\exp\left[-\frac{1}{T_{\alpha }}{\hat{S}}_{\alpha }{\hat{H}}_{B,\alpha }{\hat{S}}_{\alpha }^{†}\right],\\ {\hat{S}}_{\alpha }:=\prod_{k}\exp\left[\frac{1}{2}\lambda_{\alpha k}^{*}{\hat{b}}_{\alpha k}^{2}-h.c.\right],\;\lambda_{\alpha k}=r_{\alpha k}e^{-i\theta_{\alpha k}}, \end{array}$$

where ${\hat{S}}_{\alpha }$ is the squeezing operator for bath-α. Below we will use the master equation to calculate the Spohn formula (18), and compare it with the result by directly calculating the bath entropy change. We will see, in this non-thermal case, the Spohn formula (18) is still equal to the increasing rate of the correlation between the system and the squeezed baths.

#### 2.4.1. Master Equation

We first look at the dynamics of the open system alone. The total s + b system follows the von Neumann equation $\partial_{t}{\hat{\rho }}_{\mathrm{SB}}\left(t\right)=i\left[{\hat{\rho }}_{\mathrm{SB}}\left(t\right),\;{\hat{H}}_{S+B}\right]$. Based on it, after the Born-Markovian approximation [38,52], we can derive a master equation ${\dot{\hat{\rho }}}_{S}=\sum_{\alpha }L_{\alpha }\left[{\hat{\rho }}_{S}\right]$ for the open system ${\hat{\rho }}_{S}\left(t\right)$ (interaction picture), where (see the detailed derivation in Reference [16])

(20) $$\begin{array}{cc} L_{\alpha }\left[{\hat{\rho }}_{S}\right]=\gamma_{\alpha } & [n_{\alpha }\left({\hat{a}}^{†}{\hat{\rho }}_{S}\hat{a}-\frac{1}{2}\left\{\hat{a}{\hat{a}}^{†},{\hat{\rho }}_{S}\right\}\right)+\left(n_{\alpha }+1\right)\left(\hat{a}{\hat{\rho }}_{S}{\hat{a}}^{†}-\frac{1}{2}\left\{{\hat{a}}^{†}\hat{a},{\hat{\rho }}_{S}\right\}\right)\\ & -u_{\alpha }\left({\hat{a}}^{†}{\hat{\rho }}_{S}{\hat{a}}^{†}-\frac{1}{2}\left\{{\left({\hat{a}}^{†}\right)}^{2},{\hat{\rho }}_{S}\right\}\right)-u_{\alpha }^{*}\left(\hat{a}{\hat{\rho }}_{S}\hat{a}-\frac{1}{2}\left\{{\hat{a}}^{2},{\hat{\rho }}_{S}\right\}\right)]. \end{array}$$

Here $n_{\alpha }:=\left({\bar{n}}_{\alpha ,\Omega }+\frac{1}{2}\right)\cosh2r_{\alpha \Omega }-\frac{1}{2}$, $u_{\alpha }:=e^{i\theta_{\alpha \Omega }}\left({\bar{n}}_{\alpha ,\Omega }+\frac{1}{2}\right)\sinh2r_{\alpha \Omega }$. The parameters $r_{\alpha \Omega }$ and $\theta_{\alpha \Omega }$ take values from $\lambda_{\alpha k}$ in Equation (19) when $\omega_{k}\to \Omega$, and ${\bar{n}}_{\alpha ,\Omega }:=1/\left[\exp\left(\Omega /T_{\alpha }\right)-1\right]$ is the Planck function. The decay factor $\gamma_{\alpha }:=J_{\alpha }\left(\Omega \right)=K_{\alpha }\left(\Omega \right)$ is defined from the coupling spectrums $J_{\alpha }\left(\omega \right):=2\pi \sum_{k}{\left|g_{\alpha k}\right|}^{2}\delta \left(\omega -\omega_{\alpha k}\right)$ and $K_{\alpha }\left(\omega \right):=2\pi \sum_{k}g_{\alpha k}^{2}\delta \left(\omega -\omega_{\alpha k}\right)$. In addition, we have omitted the phase of $g_{\alpha k}$, thus $K_{\alpha }\left(\omega \right)=K_{\alpha }^{*}\left(\omega \right)=J_{\alpha }\left(\omega \right)$. From this master equation, we obtain

(21) $$\begin{array}{c} \frac{d}{dt}\langle \tilde{a}\left(t\right)\rangle =-\sum_{\alpha }\frac{1}{2}\gamma_{\alpha }\langle \tilde{a}\rangle ,\;\frac{d}{dt}\langle {\tilde{a}}^{2}\rangle =-\sum_{\alpha }\gamma_{\alpha }\left[\langle {\tilde{a}}^{2}\rangle -u_{\alpha }\right],\\ \frac{d}{dt}\langle {\tilde{a}}^{†}\tilde{a}\rangle =-\sum_{\alpha }\gamma_{\alpha }\left[\langle {\tilde{a}}^{†}\tilde{a}\rangle -n_{\alpha }\right]. \end{array}$$

Here ${\hat{\rho }}_{S}\left(t\right)$ is in the interaction picture, $\langle \tilde{o}\left(t\right)\rangle :=\mathrm{tr}\left[{\hat{\rho }}_{S}\left(t\right)\hat{o}\right]$, and $\hat{o}$ is in the Schrödinger picture.

For this master equation, the partial steady state ${\hat{\rho }}_{\mathrm{SS}}^{\left(\alpha \right)}$ associated with bath-α, which satisfies $L_{\alpha }\left[{\hat{\rho }}_{\mathrm{SS}}^{\left(\alpha \right)}\right]=0$, is a squeezed thermal one,

(22) $$\begin{array}{c} {\hat{\rho }}_{\mathrm{SS}}^{\left(\alpha \right)}=\frac{1}{z_{\alpha }}\exp\left[-\frac{\Omega }{T_{\alpha }}\cdot {\hat{S}}_{\alpha }{\hat{a}}^{†}\hat{a}{\hat{S}}_{\alpha }^{†}\right],\\ {\hat{S}}_{\alpha }:=\exp\left[-\left(\frac{1}{2}\zeta_{\alpha }^{*}{\hat{a}}^{2}-h.c.\right)\right],\;\zeta_{\alpha }=\lambda_{\alpha k}{|}_{\omega_{k}=\Omega }:=r_{\alpha }e^{i\theta_{\alpha }}, \end{array}$$

where ${\hat{S}}_{\alpha }$ is the squeezing operator. Now we can put this result into the Spohn formula (18), then the term $\chi_{\alpha }=\mathrm{tr}\left[L_{\alpha }\left[\hat{\rho }\right]\cdot \ln{\hat{\rho }}_{\mathrm{SS}}^{\left(\alpha \right)}\right]$ gives

(23) $$\chi_{\alpha }=\frac{\Omega }{T_{\alpha }}\cdot \gamma_{\alpha }\left(\cosh2r_{\alpha }\cdot \left[\langle {\tilde{a}}^{†}\tilde{a}\rangle -n_{\alpha }\right]-\frac{1}{2}\sinh2r_{\alpha }\left[e^{-i\theta_{\alpha }}\left(\langle {\tilde{a}}^{2}\left(t\right)\rangle -u_{\alpha }\right)+h.c.\right]\right).$$

When there is no squeezing ($r_{\alpha }=0$), this equation exactly returns to the thermal bath result $\chi_{\alpha }=-\frac{1}{T_{\alpha }}\frac{d}{dt}\left[\Omega \langle {\tilde{a}}^{†}\tilde{a}\rangle \right]=-{\dot{Q}}_{\alpha }/T_{\alpha }$ (see Equation (21)), which is the exchange of the thermal entropy. However, due to the quantum squeezing in the bath, clearly this $\chi_{\alpha }$ term is no longer the thermal entropy, and now it looks too complicated to tell its physical meaning. Below, we are going to show that here this $\chi_{\alpha }$ term is just the informational entropy changing of bath-α.

#### 2.4.2. Bath Entropy Dynamics

Now we calculate the entropy change ${\dot{S}}_{B,\alpha }$ of bath-α directly by adopting the similar approximation as Equation (9), and that gives

(24) $$\begin{array}{cc} {\dot{S}}_{B,\alpha } & ≃-\mathrm{tr}\left[{\dot{\hat{\rho }}}_{B,\alpha }\left(t\right)\ln\left(\frac{1}{Z_{\alpha }}\exp\left[-\frac{1}{T_{\alpha }}\;{\hat{S}}_{\alpha }{\hat{H}}_{B,\alpha }{\hat{S}}_{\alpha }^{†}\right]\right)\right]\\ & =\sum_{k}\frac{\omega_{\alpha k}}{T_{\alpha }}\left(\cosh2r_{\alpha k}\cdot \frac{d}{dt}\langle {\tilde{b}}_{\alpha k}^{†}\left(t\right){\tilde{b}}_{\alpha k}\left(t\right)\rangle +\frac{1}{2}\sinh2r_{\alpha k}\left[e^{-i\theta_{\alpha k}}\cdot \frac{d}{dt}\langle {\tilde{b}}_{\alpha k}^{2}\left(t\right)\rangle +h.c.\right]\right). \end{array}$$

Unlike the thermal baths case in Equation (9), here it is not easy to see how the bath entropy dynamics ${\dot{S}}_{B,\alpha }$ is related the system dynamics. However, notice that ${\dot{S}}_{B,\alpha }$ is simply determined by the time derivative of the bath operator expectations like $\langle {\tilde{b}}_{\alpha k}^{†}\left(t\right){\tilde{b}}_{\alpha k}\left(t\right)\rangle$ and $\langle {\tilde{b}}_{\alpha k}^{2}\left(t\right)\rangle$, which can be further calculated by Heisenberg equations. After certain Markovian approximation, in the weak coupling limit ($\gamma_{\alpha }\ll \Omega$), we can prove the following relation (see the detailed proof in Reference [16]),

(25) $$\begin{array}{cc} \sum_{k}f_{k}\cdot \frac{d}{dt}\langle {\tilde{b}}_{\alpha k}^{†}{\tilde{b}}_{\alpha k}\rangle & ≃f\left(\omega_{k}\to \Omega \right)\cdot \gamma_{\alpha }\left[\langle {\tilde{a}}^{†}\tilde{a}\rangle -n_{\alpha }\right]=-\mathrm{tr}\left\{f\left(\Omega \right){\tilde{a}}^{†}\tilde{a}\cdot L_{\alpha }\left[\rho \right]\right\},\\ \sum_{k}h_{k}\cdot \frac{d}{dt}\langle {\tilde{b}}_{\alpha k}^{2}\rangle & ≃-h\left(\omega_{k}\to \Omega \right)\cdot \gamma_{\alpha }\left[\langle {\tilde{a}}^{2}\rangle -u_{\alpha }\right]=\mathrm{tr}\left\{h\left(\Omega \right){\tilde{a}}^{2}\cdot L_{\alpha }\left[\rho \right]\right\}, \end{array}$$

where $f_{k}$ and $h_{k}$ are the summation weights associated with the bath mode ${\hat{b}}_{\alpha k}$.

These two relations well connects the dynamics of bath-α (left sides) with that of the open system (right sides). For example, let $f_{k}=\omega_{\alpha k}$, then the above relation becomes $\frac{d}{dt}\left[\sum_{k}\omega_{\alpha k}\langle {\tilde{b}}_{\alpha k}^{†}{\tilde{b}}_{\alpha k}\rangle \right]≃-\mathrm{tr}\left(\Omega {\tilde{a}}^{†}\tilde{a}\cdot L_{\alpha }\left[\rho \right]\right)$, which is just the heat emission-absorption relation $\frac{d}{dt}\langle {\hat{H}}_{B,\alpha }\rangle =-{\dot{Q}}_{\alpha }$, and we have utilized it in the discussion below Equation (9). To calculate the above entropy change Equation (24) for the squeezed thermal bath, let $f_{k}=\frac{1}{T_{\alpha }}\omega_{\alpha k}\cosh2r_{\alpha k}$, $h_{k}=\frac{1}{2T_{\alpha }}\omega_{\alpha k}e^{-i\theta_{\alpha k}}\sinh2r_{\alpha k}$, then we obtain

(26) $${\dot{S}}_{B,\alpha }=\frac{\Omega }{T_{\alpha }}\cdot \gamma_{\alpha }\left(\cosh2r_{\alpha }\cdot \left[\langle {\tilde{a}}^{†}\tilde{a}\rangle -n_{\alpha }\right]-\frac{1}{2}\sinh2r_{\alpha }\left[e^{-i\theta_{\alpha }}\left(\langle {\tilde{a}}^{2}\left(t\right)\rangle -u_{\alpha }\right)+h.c.\right]\right).$$

This result exactly equals to the term $\chi_{\alpha }=\mathrm{tr}\left[L_{\alpha }\left[\rho \right]\ln\rho_{\mathrm{SS}}^{\left(\alpha \right)}\right]$ in the above Spohn formula (see Equation (23)). Therefore, in this non-thermal bath case, the changing rate of the system-bath mutual information is just equal to the Spohn formula (18),

(27) $$\frac{d}{dt}I_{\mathrm{SB}}={\dot{S}}_{S}+\sum_{\alpha }{\dot{S}}_{B,\alpha }=R_{\mathrm{Sp}}\geq 0,$$

thus its positivity is still guaranteed [16].

That means that although the non-thermal baths are beyond the applicable scope of standard thermodynamics, namely, $dS_{i}=dS-\sum_{\alpha }đQ_{\alpha }/T_{\alpha }\geq 0$ does not apply, the system-bath correlation $I_{\mathrm{SB}}$ still keeps increasing monotonically like in the thermal bath case (Section 2.2). Therefore, this system-bath correlation production may be a generalization for the irreversible entropy production which also applies for the non-thermal cases. Tracing back to the original consideration of the irreversible entropy change (Equation (3)), it turns out the term $-dS_{e}$ can also be regarded the informational entropy change of the bath, and it gives the relation $dS_{e}=đQ/T$ in the special case of canonical thermal bath.

### 2.5. Discussions

Historically, the Spohn formula was first introduced by considering the distance between the system state $\hat{\rho }\left(t\right)$ and its final steady state ${\hat{\rho }}_{\mathrm{SS}}$, which is measured by their relative entropy $S\left[\hat{\rho }\left(t\right)∥{\hat{\rho }}_{\mathrm{SS}}\right]:=-\mathrm{tr}\left[\hat{\rho }\left(t\right)\cdot \left(\ln\hat{\rho }\left(t\right)-\ln{\hat{\rho }}_{\mathrm{SS}}\right)\right]$ (Spohn [42]). When t→∞, $\hat{\rho }\left(t\right)\to {\hat{\rho }}_{\mathrm{SS}}$, and this distance decreases to zero. Thus, for a Markovian master equation $\partial_{t}\hat{\rho }=L\left[\hat{\rho }\right]$, the EPr is defined from the time derivative of this distance, i.e.,

(28) $$\sigma :=-\frac{d}{dt}S\left[\hat{\rho }\left(t\right)∥{\hat{\rho }}_{\mathrm{SS}}\right]=\mathrm{tr}\left[\left(\ln{\hat{\rho }}_{\mathrm{SS}}-\ln\hat{\rho }\right)L\left[\hat{\rho }\right]\right].$$

Therefore, this EPr-σ serves as a Lyapunov index for the master equation. It was proved that σ≥0, and σ=0 when t→∞. When there is only one thermal bath, this EPr-σ returns to the thermodynamics result, $\sigma =\dot{S}-\dot{Q}/T$.

However, when the open system contacts with multiple heat baths as described by the master Equation (17), denoting $L\left[\hat{\rho }\right]=\sum_{\alpha }L_{\alpha }\left[\hat{\rho }\right]$, the above EPr-σ still goes to zero when t→∞. Thus it does not tell the difference between achieving the equilibrium state or the stationary non-equilibrium state (see the example of Equation (6)). Later (Spohn, Lebowitz [43]), this EPr-σ was generalized to be the form of Equations (7) and (18), and its positivity can be proved by the similar procedure given in the previous study (Spohn [42]).

In standard thermodynamics, the second law statement $dS_{i}\geq 0$ requires a monotonic increase of the irreversible entropy, not only compared with the initial state. Notice that in the proof for the positivity of the EPr, the Markovianity is necessary for both classical and quantum cases. If the master equation of the open system is non-Markovian, it is possible that there exist certain periods where $R_{\mathrm{EP}}<0$, which means the decrease of the irreversible entropy (or the system-bath correlation). When comparing EPr with standard thermodynamics, a coarse-grained time scale is more proper (which usually means the Markovian process), thus $R_{\mathrm{EP}}<0$ may be acceptable if it appears only in short time scales.

In the above discussions, clearly the most important part is how to calculate the bath entropy dynamics directly. This is usually quite difficult since the bath contains infinite number of DoF. The above calculation can be done mainly thanks to the approximation ${\dot{S}}_{B}≃-\mathrm{tr}\left[{\dot{\hat{\rho }}}_{B}\left(t\right)\ln{\hat{\rho }}_{B}\left(0\right)\right]$. The results derived thereafter are consistent with the previous conclusions in thermodynamics, but still we need more examination about the validity of this approximation.

There are a few models of open system that are exactly solvable for this examination. In Reference [23], the bath entropy dynamics was calculated when a two-level-system (TLS) is dispersively coupled with a squeezed thermal bath. In this problem, the density matrix evolution of each bath mode can be exactly solved. The state of each bath mode is the probabilistic summation of two displaced Gaussian states ${\hat{\varrho }}_{k}\left(t\right)=p_{+}{\hat{\varrho }}_{k}^{+}\left(t\right)+p_{-}{\hat{\varrho }}_{k}^{-}\left(t\right)$, which keep separating and recombining periodically in the phase space. Thus the exact entropy dynamics can be calculated and compared with the result based on the above approximation.

It turns out that the above approximation fits the exact result quite well in the high temperature area; in the low temperature area, the approximated result diverges to infinity when T→0, but the exact result remains finite. This is because in the high temperature area, this ${\hat{\varrho }}_{k}\left(t\right)$ can be better approximated as a single Gaussian state when the separation of ${\hat{\varrho }}_{k}^{\pm }\left(t\right)$ is quite small; while in the low temperature area, the uncertainty of ${\hat{\varrho }}_{k}\left(t\right)$ mainly comes from the probabilities $p_{\pm }$ but not the entropy in the Gaussian states ${\hat{\varrho }}_{k}^{\pm }$. Namely, in the low temperature area, the influence from the system to the bath is bigger, especially for nonlinear systems like the TLS. If the bath states cannot be well treated as Gaussian ones, the above approximation is questionable, and how to calculate the bath entropy in this case remains an open problem.

## 3. The Entropy in the Ideal Gas Diffusion

In the above discussions about the correlation production between the open system and its environment, we utilized an important condition, i.e., the whole s + b system is an isolated system, thus its entropy does not change with time. In the quantum case, the whole s + b system follows the von Neumann equation $\partial_{t}{\hat{\rho }}_{\mathrm{SB}}=i\left[{\hat{\rho }}_{\mathrm{SB}},\;{\hat{H}}_{S+B}\right]$, thus the von Neumann entropy $S_{V}\left[{\hat{\rho }}_{\mathrm{SB}}\right]=-\mathrm{tr}\left[{\hat{\rho }}_{\mathrm{SB}}\ln{\hat{\rho }}_{\mathrm{SB}}\right]$ does not change during the unitary evolution. Likewise, in the classical case, the whole system follows the Liouville equation $\partial_{t}\rho_{\mathrm{SB}}=-\left\{\rho_{\mathrm{SB}},\;H_{S+B}\right\}$, thus the Gibbs entropy $S_{G}\left[\rho \right]=-\sum_{n}p_{n}\ln p_{n}$ keeps a constant.

However, still this is quite counter-intuitive compared with our intuition of the macroscopic irreversibility. For example, considering the diffusion process of an ideal gas as we mentioned in the very beginning (Figure 1), although there are no particle interactions and the dynamics of the whole system is well predictable, still we could see the diffusion proceeds irreversibly, and would finally occupy the whole volume uniformly.

In this section, we will show this puzzle also can be understood in the sense of correlation production. In open systems, we have seen it is the system-bath correlation that increases, while the total s + b entropy does not change. In an isolated system, there is no partition for “system” and “bath”, but we will see it is the correlation between different DoF, e.g., position-momentum, and particle-particle, that increases monotonically, while the total entropy does not change [4,27].

### 3.1. Liouville Dynamics of the Ideal Gas Diffusion

Here we make a full calculation on the phase-space evolution of the above ideal gas diffusion process in classical physics, so as to examine the dynamical behavior of the microstate, as well as its entropy.

Since there is no interaction between particles, the dynamics of the 3N DoF are independent from each other. Assuming there is no initial correlation between different DoF, the total *N*-particle microstate PDF always can be written as a product form, i.e., $\rho \left(\vec{P},\vec{Q},t\right)=\prod_{i,\sigma }\varrho \left(p_{i}^{\sigma },q_{i}^{\sigma },t\right)$ for σ=x,y,z, thus this problem can be reduced to study the PDF of a single DoF ϱ(p,x,t). Correspondingly, the Liouville equation is

(29) $$\partial_{t}\varrho =-\left\{\varrho ,H\right\}=-\frac{\partial \varrho }{\partial x}\frac{\partial H}{\partial p}+\frac{\partial \varrho }{\partial p}\frac{\partial H}{\partial x}=-\frac{p}{m}\partial_{x}\varrho ,$$

where $H=p^{2}/2m$ is the single DoF Hamiltonian.

This equation is exactly solvable, and the general solution is $\Phi (p,x-\frac{p}{m}t)$. The detailed form of the function Φ(⋯,⋯) should be further determined by the initial and boundary conditions. We assume initially the system starts from an equilibrium state confined in the area x∈[a,b], namely

(30) ϱ(p,x,0)=Λ(p)×Π(x).

**Figure 3:**
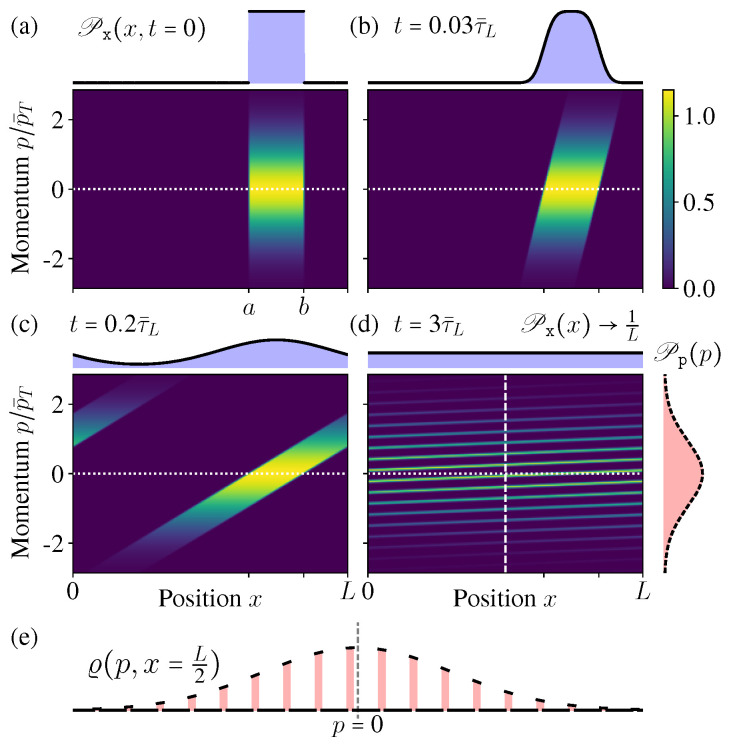
(**a**–**d**) The distribution ϱ(p,x,t) in phase space at different times (${\bar{\tau }}_{L}:=mL/{\bar{p}}_{T}$ as the time unit). As the time increases, $P_{p}\left(p\right)$ does not change, but $P_{x}\left(x,t\right)$ approaches the new uniform distribution in x∈[0,L]. (**e**) The conditional distribution at a fixed position ϱ(p,x=L/2) (vertical dashed line in (**d**)).

Here $\Lambda \left(p\right)=\frac{1}{Z}\exp\left[-p^{2}/2{\bar{p}}_{T}^{2}\right]$ is the MB distribution, with ${\bar{p}}_{T}^{2}/2m=\frac{1}{2}k_{B}T$ as the average kinetic energy, and $Z=\sqrt{2\pi }\;{\bar{p}}_{T}$ is a normalization factor. Π(x) is the initial spatial distribution (Figure 3a)

(31) $$\Pi \left(x\right)=\left\{\begin{array}{cc} \frac{1}{b-a}, & a\leq x\leq b,\\ 0, & \mathrm{elsewhere}. \end{array}\right.$$

Such a product form of ϱ(p,x,0) indicates the spatial and momentum distributions have no correlations a priori.

For the diffusion in free space x∈(−∞,∞), the time-dependent solution is

(32) $$\varrho_{F}\left(p,x,t\right)=\Lambda \left(p\right)\Pi \left(x-\frac{p}{m}t\right),$$

which satisfies both the Liouville Equation (29) and the initial condition (30).

For a confined area x∈[0,L] with periodic boundary condition ϱ(p,0,t)=ϱ(p,L,t), the solution can be constructed with the help of the above free space one, i.e.,

(33) $$\varrho \left(p,x,t\right)=\sum_{n=-\infty }^{\infty }\varrho_{F}\left(p,x+nL,t\right),\;0\leq x\leq L.$$

**Figure 4:**
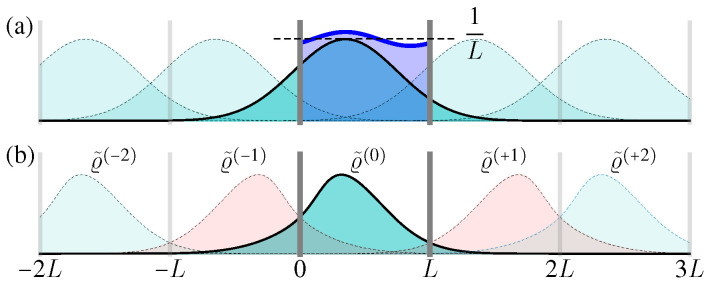
Demonstration for how the solutions are constructed for (**a**) periodic and (**b**) reflecting boundary conditions. The free space is cut into intervals of length *L*, and each contributes an “image” source. The solution is all their summation in x∈[0,L] (blue line in (**a**)).

Here $\varrho_{F}\left(p,x+nL,t\right)$ can be regarded as the periodic “image” solution in the interval [nL,nL+L] (Figure 4a) [35]. Clearly, Equation (33) satisfies the periodic boundary condition, as well as the initial condition (30), and it is simple to verify each summation term satisfies the above Liouville Equation (29), thus Equation (33) describes the full microstate PDF evolution in the confined area x∈[0,L] with periodic boundary condition.

From these exact solutions (32) and (33), it is clear to see the microstate PDF ϱ(p,x,t) can no longer hold the separable form like $f_{x}\left(x,t\right)\times f_{p}\left(p,t\right)$ once the diffusion starts, thus indeed it is not evolving towards any equilibrium state, since an equilibrium state must have a separable form similar like the initial condition (30) (see also Figure 3a).

In Figure 3 we show the microstate PDF ϱ(p,x,t) at different times. As the time increases, the “stripe” in Figure 3a becomes more and more inclined; once exceeding the boundary, it winds back from the other side due to the periodic boundary condition and generates a new “stripe” (Figure 3c). After very long time, more and more stripes appear, much denser and thinner, but they would never occupy the whole phase space continuously (Figure 3d,e).

Figure 3e shows the conditional PDF of the momentum when the position is fixed at x=L/2 (the vertical dashed line in Figure 3d). In the limit t→∞, it becomes an exotic function discontinuous everywhere, but not the MB distribution. All these features indicate that, during this diffusion process of the isolated ideal gas, indeed the ensemble is not evolving towards the new equilibrium state as expected in the macroscopic intuition. Even after long time relaxation, the microstate PDF ϱ(p,x,t→∞) is not approaching the equilibrium state.

### 3.2. Spatial and Momentum Distributions

Even after long time relaxation, the ideal gas would not achieve the new equilibrium state. This result looks counter-intuitive, since clearly we can see the particles spread all over the box uniformly after long enough time relaxation. However, we must notice that the fact “spreading all over the box uniformly” is implicitly focused on the position distribution $P_{x}\left(x,t\right)$ alone, but not the whole ensemble state ϱ(p,x,t). As a marginal distribution of ϱ(p,x,t), the spatial distribution $P_{x}\left(x,t\right)\to 1/L$ does approach the new uniform one as its steady state (Figure 3d), and now we show indeed this is true for any initial state of Π(x) [4,27].

We first consider the initial spatial distribution is a δ-function concentrated at $x_{0}$, $\Pi \left(x\right)=\delta \left(x-x_{0}\right)$. Since we have obtained the analytical results (32) and (33) for the ensemble evolution ϱ(p,x,t), the spatial distribution $P_{x}\left(x,t\right)$ emerges as its marginal distribution by averaging over the momentum:

(34) $$\begin{array}{cc} P_{x}\left(x,t\right) & =\int_{-\infty }^{\infty }dp\;\varrho \left(p,x,t\right)=\int_{-\infty }^{\infty }dp\;\sum_{n=-\infty }^{\infty }\frac{1}{Z}\exp\left[-\frac{p^{2}}{2{\bar{p}}_{T}^{2}}\right]\times \delta \left(x+nL-x_{0}-\frac{p}{m}t\right)\\ & =\sum_{n=-\infty }^{\infty }\frac{m}{Zt}\exp\left[-\frac{1}{2{\bar{v}}_{T}^{2}t^{2}}{\left(x+nL-x_{0}\right)}^{2}\right], \end{array}$$

where ${\bar{v}}_{T}:={\bar{p}}_{T}/m$. With the increase of time *t*, these Gaussian terms becomes wider and lower (Figure 4a). Therefore, when t→∞, the spatial distribution $P_{x}\left(x,t\right)$ always approaches the uniform distribution in x∈[0,L].

Any initial spatial distribution can be regarded as certain combination of δ-functions, i.e., $\Pi \left(x\right)=\int dx_{0}\Pi \left(x_{0}\right)\delta \left(x-x_{0}\right)$. Therefore, for any initial Π(x), the spatial distribution $P_{x}\left(x,t\right)$ always approaches the uniform one as its steady state. In this sense, although the underlying Liouville dynamics obeys the time-reversal symmetry, the “irreversible” diffusion appears into our sight.

On the other hand, $P_{p}\left(p,t\right)$ never changes with time, and always maintains its initial distribution, which can be proved by simply changing the integral variable:

(35) $$\begin{array}{cc} P_{p}\left(p,t\right) & =\int_{0}^{L}dx\;\sum_{n=-\infty }^{\infty }\Lambda \left(p\right)\Pi \left(x+nL-\frac{p}{m}t\right)\\ & =\int_{-\infty }^{\infty }dx\;\Lambda \left(p\right)\Pi \left(x-\frac{p}{m}t\right)=\Lambda \left(p\right). \end{array}$$

This is all because of the periodic boundary condition, and the particles always move freely. If the particles can be reflected back at the boundaries, this momentum distribution would also change with time.

### 3.3. Reflecting Boundary Condition

Now we consider the reflecting boundary condition. In this case, when the particles hit the boundaries at x=0,L, their positions do not change, but their momentum should be suddenly changed from *p* to −p. Correspondingly, the analytical result for the ensemble evolution can be obtained by summing up the “reflection images” (Figure 4b), i.e.,

(36) $$\begin{array}{c} \varrho \left(p,x,t\right)={\tilde{\varrho }}^{\left(0\right)}\left(p,x,t\right)+\sum_{n=1}^{\infty }{\tilde{\varrho }}^{\left(-n\right)}\left(p,x,t\right)+{\tilde{\varrho }}^{\left(+n\right)}\left(p,x,t\right),\;\mathrm{for}\;x\in \left[0,L\right],\\ {\tilde{\varrho }}^{\left(-n\right)}\left(p,x,t\right):=R_{0}\left[{\tilde{\varrho }}^{\left[+(n-1)\right]}\left(-p,x,t\right)\right],\;{\tilde{\varrho }}^{\left(+n\right)}\left(p,x,t\right):=R_{L}\left[{\tilde{\varrho }}^{\left[-(n-1)\right]}\left(-p,x,t\right)\right]. \end{array}$$

Here ${\tilde{\varrho }}^{\left(0\right)}\left(p,x,t\right)=\varrho_{F}\left(p,x,t\right)$, and $R_{a}\left[f\left(p,x\right)\right]:=f\left(p,2a-x\right)$ means making a mirror reflection to the function f(p,x) along the axis x=a. Clearly, each summation term ${\tilde{\varrho }}^{\left(-n\right)}$ can be regarded as shifted from $\varrho_{F}\left(p,x,t\right)$ or its mirror reflection, thus they all satisfy the differential relation in the Liouville Equation (29).

To verify the boundary condition, consider a diffusing distribution in x∈[0,L], initially described by ${\tilde{\varrho }}^{\left(0\right)}\left(p,x,t\right)$. As the time increases, it diffuses wider and even exceeds the box range [0,L]. The exceeded part should be reflected at the boundaries x=0,L as the next order ${\tilde{\varrho }}^{\left(\pm 1\right)}$, and added back to the total result ϱ(p,x,t). This procedure should be done iteratively, namely, when the term ${\tilde{\varrho }}^{\left(\pm n\right)}$ exceeds the boundaries, it generates the reflected term ${\tilde{\varrho }}^{\left[\mp (n+1)\right]}$ as the next summation order (Figure 4b).

Therefore, this result is quite similar to the above periodic case, except reflection should be made to certain summation terms. Based on the same reason as above, each summation term becomes more and more flat during the diffusion, thus the spatial distribution $P_{x}\left(x,t\right)$ always approaches the new uniform one as its steady state [4].

**Figure 5:**
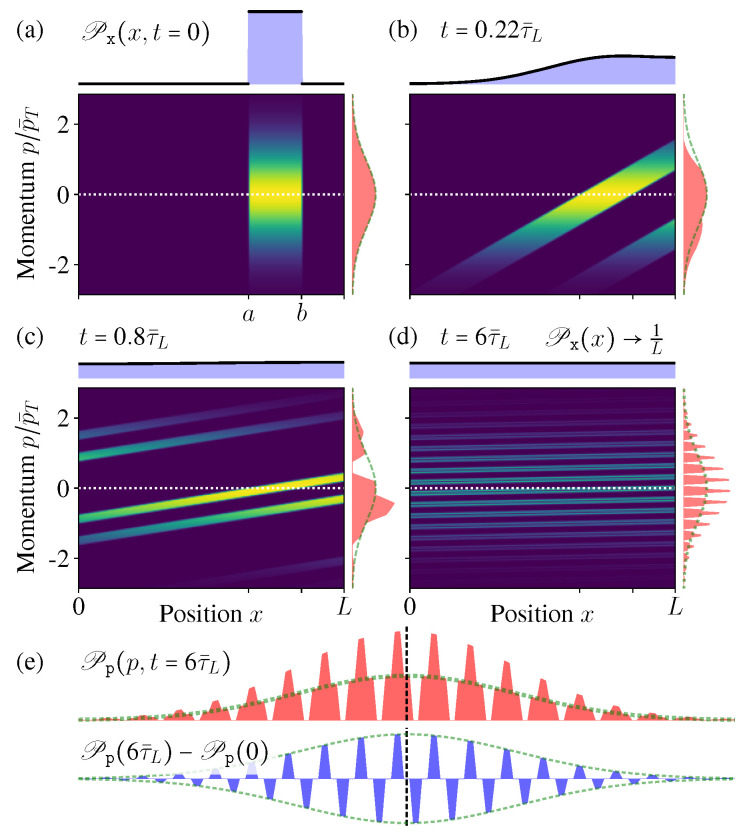
(**a**–**d**) ϱ(p,x,t) under reflecting boundary condition, as well as its spatial and momentum distributions. (**e**) The momentum distribution from (**d**), and its difference (lower blue) with the initial MB one (green dashed lines).

The ensemble evolution is shown in Figure 5a–d, which is quite similar with the above periodic case. Again, ϱ(p,x,t) is indeed not evolving towards the equilibrium state. A significant difference is the momentum distribution $P_{p}\left(p,t\right)$ now varies with time. This is because the collision at the boundaries changes the momentum direction, thus 〈p〉 is no longer conserved, although the kinetic energy $\langle p^{2}\rangle$ does not change (as the momentum amplitude).

Notice that the reflection “moves” the probability of momentum *p* to the area of −p, therefore, we see that some “areas” of $P_{p}\left(p,t\right)$ are “cut” off from the initial MB distribution, and “added” to its mirror position along p=0 (especially Figure 5c,d). Thus $P_{p}\left(p,t\right)$ is different from the initial MB distribution.

Since the reflection transfers the probability of *p* to its mirror position −p, the difference $\delta P_{p}\left(t\right):=P_{p}\left(t\right)-P_{p}\left(0\right)$ is always an odd function (lower blue in Figure 5e). As a result, the even moments $\langle p^{2n}\rangle$ of $P_{p}\left(t\right)$ are the same with the MB distribution, but the odd ones $\langle p^{2n+1}\rangle$ are changed.

**Figure 6:**
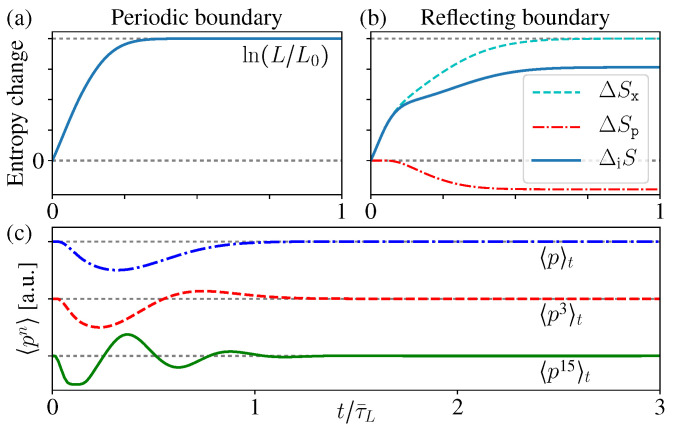
The increase of the correlation entropy $\Delta_{i}S$ for the (**a**) periodic and (**b**) reflecting boundary cases. (**c**) The evolution of odd moments ${\langle p^{n}\rangle }_{t}$ under reflecting boundary condition (the values are normalized by their maximum amplitudes for comparison). The unit ${\bar{\tau }}_{L}:=mL/{\bar{p}}_{T}$ is the time for a particle with average kinetic energy ${\bar{p}}_{T}^{2}/2m$ to pass *L*.

As the time increases, more and more “stripes” appear in $P_{p}\left(p,t\right)$, much thinner and denser. As a result, when calculating the odd orders $\langle p^{2n+1}\rangle$, the contributions from the nearest two stripes in $\delta P_{p}\left(t\right)$ (who have similar *p* values), positive and negative, tends to cancel each other (lower blue in Figure 5e). Therefore, in the limit t→∞, the odd orders $\langle p^{2n+1}\rangle$ also approach the same value of the original MB distribution (zero) (Figure 6c).

Therefore, in the long time limit, $P_{p}\left(p,t\to \infty \right)$ approaches an exotic function discontinuous everywhere, which is different from the initial MB distribution, but all of its moments $\langle p^{n}\rangle$ have the same values as the initial MB distribution [53,54]. In usual experiments, practically it is difficult to tell the difference of these two different distributions [9].

### 3.4. Correlation Entropy

From the above exact results for the ensemble evolution, we have seen that the macroscopic appearance of the new uniform distribution is indeed only about the spatial distribution, which just reflects the marginal information of the whole state ϱ(p,x,t), thus this macroscopic appearance is not enough to conclude whether ϱ(p,x,t) is approaching the new equilibrium state with entropy increase.

However, in practical experiments, the full joint distribution ϱ(p,x) is difficult to be measured directly. Usually it is the spatial and momentum distributions $P_{x}\left(x\right)$ and $P_{p}\left(p\right)$ that are directly accessible for measurements, e.g., by measuring the gas density and pressure. Therefore, based on these two marginal distributions, we may “infer” the microstate PDF as [7,25,55]

(37) $${\tilde{\varrho }}_{\inf}\left(p,x,t\right):=P_{x}\left(x,t\right)\times P_{p}\left(p,t\right),$$

which indeed neglected the correlation between these two marginal distributions. As a result, in the long time limit, $P_{x}\left(x,t\right)\to 1/L$ approaches the new uniform distribution, while $P_{p}\left(x,t\right)$ “behaves” similarly like the initial MB distribution, thus this inferred state ${\tilde{\varrho }}_{\inf}\left(p,x,t\to \infty \right)$ just looks like a new “equilibrium state”.

The entropy change of this inferred state is

(38) $$\begin{array}{cc} \Delta_{i}S\left(t\right):= & S_{G}\left[{\tilde{\varrho }}_{\inf}\left(t\right)\right]-S_{G}\left[{\tilde{\varrho }}_{\inf}\left(0\right)\right]\\ = & \left\{S_{x}\left[P_{x}\left(t\right)\right]+S_{p}\left[P_{p}\left(t\right)\right]-S_{G}\left[\varrho \left(t\right)\right]\right\}-\left\{S_{x}\left[P_{x}\left(0\right)\right]+S_{p}\left[P_{p}\left(0\right)\right]-S_{G}\left[\varrho \left(0\right)\right]\right\}, \end{array}$$

where $S_{G}\left[\varrho \left(t\right)\right]=S_{G}\left[\varrho \left(0\right)\right]$ is guaranteed by the Liouville dynamics, and

(39) $$\begin{array}{cc} S_{x}\left[P_{x}\left(x,t\right)\right] & :=-\int_{0}^{L}dx\;P_{x}\left(x,t\right)\ln P_{x}\left(x,t\right),\\ S_{p}\left[P_{p}\left(p,t\right)\right] & :=-\int_{-\infty }^{\infty }dp\;P_{p}\left(p,t\right)\ln P_{p}\left(p,t\right). \end{array}$$

Notice that the term $S_{x}+S_{p}-S_{G}:=I_{\mathrm{xp}}$ in Equation (38) is just the mutual information between the marginal distributions $P_{x}\left(x,t\right)$ and $P_{p}\left(p,t\right)$, which is the measure for their correlation [36,56] (see the discussion about the entropy for continuous PDF in Appendix A).

Therefore, here $\Delta_{i}S$ just describes the correlation increase between the spatial and momentum distributions. During the diffusion process, this correlation entropy $\Delta_{i}S\left(t\right)$ increases monotonically for both periodic and reflecting boundary cases (Figure 6a,b). Notice that this is quite similar with the above discussions about open systems, namely, the total entropy does not change, while the correlation entropy increases “irreversibly” [13,14,15,16,23,24,25,29,30,31].

For the periodic boundary case, $P_{p}\left(p\right)$ does not change, and $P_{x}\left(x\right)$ approaches the uniform distribution after long time, thus the above entropy increase (38) gives $\Delta_{i}S=\ln\left(L/L_{0}\right)$, where $L_{0}:=b-a$ is the length of the initially occupied area. When considering the full *N*-particle state of the ideal gas, the corresponding inferred state is ${\tilde{\rho }}_{\inf}\left(\vec{P},\vec{Q}\right)=\prod_{i,\sigma }{\tilde{\varrho }}_{\inf}\left(p_{i}^{\sigma },q_{i}^{\sigma }\right)$, thus it gives the entropy increase as $\Delta_{i}S=N\ln\left(V/V_{0}\right)$. Notice that this result exactly reproduces the above thermodynamic entropy increase as mentioned in the beginning of this section (Figure 6a) (these conclusions still hold in the thermodynamics limit V→∞, since the system sizes always appear in ratios, e.g., $V/V_{0}$).

For the reflecting boundary case, $P_{x}\left(x\right)\to 1/L$ still holds and gives $\Delta S_{x}=\ln\left(L/L_{0}\right)$, but now $P_{p}\left(p\right)$ varies with time. Moreover, it is worthwhile to notice that $\Delta S_{p}\left[P_{p}\right]$ is decreasing with time (Figure 6b), which looks a little counter-intuitive. The reason is, as we mentioned before, the boundary reflections change the momentum directions and so as the distribution $P_{p}\left(p\right)$, but the average energy $\langle p^{2}\rangle$ does not change, thus the thermal distribution (which is the initial distribution) should have the maximum entropy [20,55]. Therefore, during the evolution, the deviation of $P_{p}\left(p,t\right)$ from the initial MB distribution leads to the decrease of its entropy $\Delta S_{p}\left[P_{p}\right]$. However, clearly this is quite difficult to be sensed in practice, and the total correlation entropy change $\Delta_{i}S=\Delta S_{x}+\Delta S_{p}$ still increases monotonically.

### 3.5. Resolution Induced Coarse-Graining

Historically, the problem of the constant entropy from the Liouville dynamics was first studied by Gibbs [3]. To understand why the entropy increases in standard thermodynamics, he noticed that, if we change the order of taking limit when calculating the “ensemble volume” (the entropy), the results are different. Usually, at a certain time *t*, the phase space is divided into many small cells with a finite volume $\varepsilon_{V}$, and we make summation from all these cells, then let the cell size $\varepsilon_{V}\to 0$. That gives the result of constant entropy. However, if we keep the cell size finite, and let the time t→∞ first, then let the cell size $\varepsilon_{V}\to 0$, that would give an increasing entropy.

This idea is now more specifically described as the “coarse-graining” [3,4,8]. The “coarse-grained” ensemble state ${\tilde{\rho }}_{c.g.}\left(\vec{P},\vec{Q}\right)$ is obtained by taking the phase-space average of the exact one $\rho (\vec{P},\vec{Q})$ over a small volume around each point $\left(\vec{P},\vec{Q}\right)$, namely,

(40) $${\tilde{\rho }}_{c.g.}\left(\vec{P},\vec{Q}\right):=\int_{\varepsilon_{V}}d^{3N}P\;d^{3N}Q\;\rho \left(\vec{P},\vec{Q}\right)/\varepsilon_{V}.$$

Here $\varepsilon_{V}$ is the small volume around the point $\left(\vec{P},\vec{Q}\right)$ in the phase space. From Figure 3d and Figure 5d, we can see after long time relaxation, the coarse-grained ${\tilde{\rho }}_{c.g.}\left(\vec{P},\vec{Q}\right)$ could approach the equilibrium state.

In practical measurements, there always exists a finite resolution limit, and that can be regarded as the physical origin of coarse-graining. When measuring a continuous PDF P(x), we should first divide the continuous area x∈[0,L] into *N* intervals, and then measure the probability that appears in the interval between $x_{n}$ and $x_{n}+\Delta x$, which is denoted as $p_{n}=P\left(x_{n}\right)\Delta x$. In the limit Δx→0, the histogram $P(x_{n})$ becomes the continuous probability density (see also Appendix A).

Here Δx is determined by the measurement resolution, but practical measurements always have a finite resolution limit $\Delta x≳\delta \tilde{x}$, thus cannot approach 0 arbitrarily. Therefore, the fine structure within the minimum resolution interval $\delta \tilde{x}$ of the continuous PDF P(x) cannot be sensed in practice. However, usually P(x) is assumed to be a smooth function within this small interval, thus indeed P(x) is coarse-grained by its average value in this small region, and the resolution limit $\delta \tilde{x}$ practically determines the coarse-graining size.

**Figure 7:**
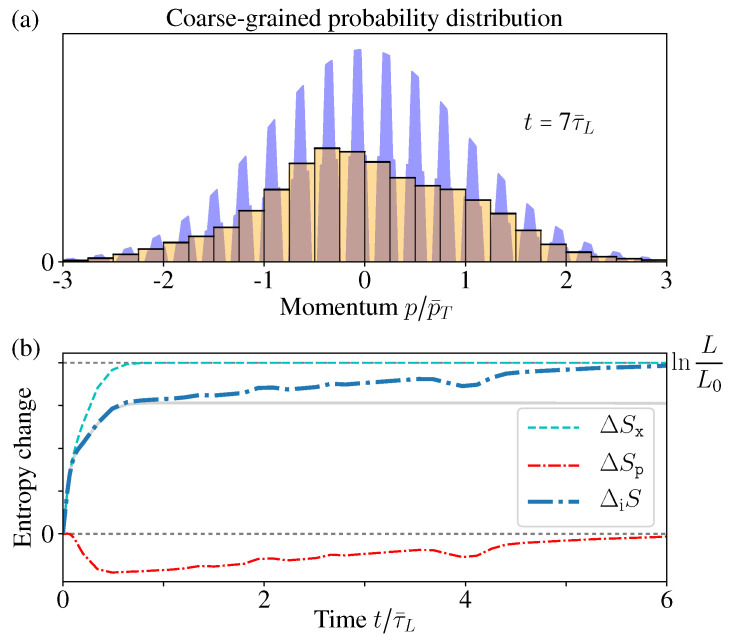
(**a**) The exact distribution $P_{p}\left(p\right)$ (blue) and its coarse-grained distribution ${\tilde{P}}_{p}\left(p\right)$ (orange histogram) at $t=7{\bar{\tau }}_{L}$, which is quite close to the MB one. (**b**) The entropy change calculated by the coarse-grained distribution ${\tilde{P}}_{p}\left(p\right)$, where the solid gray line is the entropy change calculated from the exact $P_{p}\left(p\right)$ for comparison (the same as Figure 6b). The parameters are the same with those in Figure 6. The coarse-graining size is $\delta \tilde{p}=0.2{\bar{p}}_{T}$.

Remember in the reflecting boundary case, the momentum distribution $P_{p}\left(p\right)$ approaches an exotic function with a structure of dense comb and discontinuous everywhere. Therefore, such an exotic structure within the resolution limit indeed cannot be observed in practice. For a fixed measurement resolution $\delta \tilde{p}$, there always exists a certain time $t_{\delta \tilde{p}}$, so that after $t>t_{\delta \tilde{p}}$, the “comb teeth” in Figure 7a are finer than the resolution $\delta \tilde{p}$. In addition, when $t\gg t_{\delta \tilde{p}}$, the coarse-grained distribution ${\tilde{P}}_{p}\left(p\right)$ (with coarse-graining size $\delta \tilde{p}$) would well approach the thermal distribution (Figure 7b).

In Figure 6c, we have shown that all the moments $\langle p^{n}\rangle$ of $P_{p}\left(p,t\to \infty \right)$ have no difference with the initial MB distribution. Now due to the finite resolution limit, again we have no way to tell the difference between the exotic function $P_{p}\left(p,t\to \infty \right)$ and its coarse-graining ${\tilde{P}}_{p}\left(p,t\to \infty \right)$, which goes back to the initial MB distribution (Figure 7a). In this sense, the momentum distribution $P_{p}\left(p,t\to \infty \right)$ has no “practical” difference with the thermal equilibrium distribution.

### 3.6. Entropy “Decreasing” Process

Now we see the correlation entropy, rather than the total entropy, coincides closer to the irreversible entropy increase in macroscopic thermodynamics. Here we show it could be possible, although not quite feasible in practice, to construct an “entropy decrease” process.

To achieve this, we first let the ideal gas experience the above diffusion process with “entropy increase” for a certain time $t_{*}$ (Figure 3 and Figure 5). From the state $\varrho (p,x,t_{*})$ at this moment (e.g., Figure 5d), we construct a new “initial state” by reversing its momentum $\varrho^{\prime }\left(0\right)=\varrho \left(-p,x,t_{*}\right)$. Since the Liouville equation obeys time-reversal symmetry, this new “initial state” would evolve into $\varrho^{\prime }\left(t_{*}\right)=\varrho \left(-p,x,0\right)$ after time $t_{*}$, which is just the original equilibrium state confined in x∈[a,b] (Figure 5a). That means, the idea gas exhibits a process of “reversed diffusion”.

During this time-reversal process, the total Gibbs entropy, which contains the full information, still keeps constant. However, the correlation entropy change $\Delta_{i}S$ (no matter whether coarse-grained) would exactly experience the reversed “backward” evolution of Figure 6, which is an “entropy decreasing” process.

This is just the idea of the Loschmidt paradox [8,57,58,59,60], except two subtle differences: (1) here we are talking about the ideal gas with no particle collision, but the original Loschmidt paradox was about the Boltzmann transport equation in the presence of particle collisions; (2) here we are talking about the correlation entropy between the spatial and momentum distributions, while the Boltzmann equation is about the entropy of the single-particle PDF.

We must notice that such an initial state $\varrho^{\prime }\left(0\right)$ is NOT an equilibrium state, but contains very delicate correlations between the spatial and momentum distributions [5]. To see such a time-reversal process, the initial state must be precisely prepared to contain such specific correlations between the marginal distributions (Figure 5d), which is definitely quite difficult for practical operation. Therefore, such an “entropy decrease” process is rarely seen in practice (except some special cases like the Hahn echo [61] and back-propagating wave [62,63]).

## 4. The Correlation in the Boltzmann Equation

In the above section, we focused on the diffusion of the ideal gas with no inter-particle interactions, and the initial momentum distribution has been assumed to be the MB distribution a priori. If the initial momentum distribution is not the MB one, the particles could still exhibit the irreversible diffusion filling the whole volume, but $P_{p}\left(p,t\right)$ would never become the thermal equilibrium distribution.

If there exist weak interactions between the particles, as shown by the Boltzmann *H*-theorem, the single-particle PDF f(p,r,t) could always approach the MB distribution as its steady state, together with the irreversible entropy increase. In addition, in this case, the above “coarse-graining” is not needed for f(p,r,t) to approach the thermal equilibrium distribution.

Notice that, in the Boltzmann *H*-theorem, only the single-particle PDF f(p,r,t) is concerned. The total ensemble $\rho (\vec{P},\vec{Q},t)$ in the 6N-dimensional phase space should still follow the Liouville equation, thus its entropy does not change with time. Therefore, the *H*-theorem conclusion also can be understood as the inter-particle correlations are increasing irreversibly [1,5,32]. Again, this is quite similar like the correlation understanding in the last two sections, and here the inter-particle correlation entropy could well reproduce the entropy increase result in the standard macroscopic thermodynamics.

The debates about the Boltzmann *H*-theorem started ever since its birth. The most important one must be the Loschmidt paradox raised in 1876: due to the time-reversal symmetry of the microscopic dynamics of the particles, once their momenta are reversed at the same time, the particles should follow their incoming paths “backward”, which is surely a possible evolution for the microstate; however, if the entropy of f(p,r,t) must increase (according to the *H*-theorem), then the corresponding “backward” evolution must give an entropy decreasing process, which is contradicted with the *H*-theorem conclusion.

In this section, we will see the slowly increasing inter-particle correlations could be helpful in understanding this paradox. Namely, due to the significant inter-particle correlation established during the “forward” process, indeed the “backward” process no longer satisfies the molecular-disorder assumption, which is a crucial approximation in deriving the Boltzmann equation, thus it is not suitable to be described by the Boltzmann equation, and the *H*-theorem does not apply in this case either.

### 4.1. Derivation of the Boltzmann Equation

We first briefly review the derivation of the Boltzmann transport equation [1,64,65]. When there is no external force, the evolution equation of the single-particle microstate PDF $f(p_{1},r_{1},t)$ is

(41) $$\left[\partial_{t}+\frac{p_{1}}{m}\cdot \nabla_{r_{1}}\right]f\left(p_{1},r_{1},t\right)=\partial_{t}f{|}_{\mathrm{col}}.$$

The left side is just the above Liouville Equation (29) of the ideal gas, and the right side is the probability change due to the particle collision (assuming only bipartite collisions exist).

This collision term, rewritten as $\partial_{t}f{|}_{\mathrm{col}}=\Delta^{\left(+\right)}-\Delta^{\left(-\right)}$, contains two contributions: $\Delta^{\left(-\right)}$ means the collision between two particles $\left(p_{1}r_{1};\;p_{2}r_{2}\right)\to \left(p_{1}^{\prime }r_{1}^{\prime };\;p_{2}^{\prime }r_{2}^{\prime }\right)$ kicks particle-1 out of its original region around $\left(p_{1},r_{1}\right)$, thus $f(p_{1},r_{1},t)$ decreases; likewise, $\Delta^{\left(+\right)}$ means the collision $\left(p_{1}^{\prime }r_{1}^{\prime };\;p_{2}^{\prime }r_{2}^{\prime }\right)\to \left(p_{1}r_{1};\;p_{2}r_{2}\right)$ kicks particle-1 into the region around $\left(p_{1},r_{1}\right)$ and that increases $f(p_{1},r_{1},t)$.

**Figure 8:**
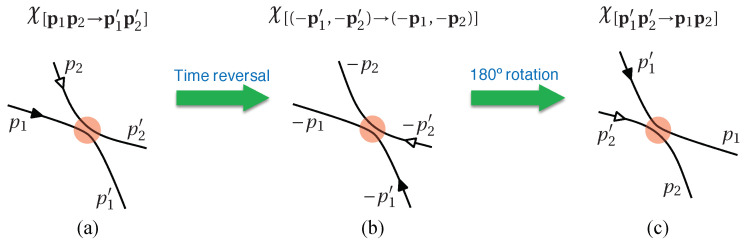
Assume collisions happen only in short range, then the transition rate $\chi_{\left[\left(p_{1}r_{1};p_{2}r_{2}\right)\to \left(p_{1}^{\prime }r_{1}^{\prime };p_{2}^{\prime }r_{2}^{\prime }\right)\right]}$ could have nonzero value only within the range $r_{1}≃r_{2}≃r_{1}^{\prime }≃r_{2}^{\prime }$. The scattering process (**b**) is the time reversal of (**a**), thus they have equal transition rates $\chi_{\left[p_{1}p_{2}\to p_{1}^{\prime }p_{2}^{\prime }\right]}=\chi_{\left[\left(-p_{1}^{\prime },-p_{2}^{\prime }\right)\to \left(-p_{1},-p_{2}\right)\right]}$. The scattering process (**c**) is obtained by making the $180^{\circ }$ inversion of (**b**), thus they also have equal transition rates $\chi_{\left[\left(-p_{1}^{\prime },-p_{2}^{\prime }\right)\to \left(-p_{1},-p_{2}\right)\right]}=\chi_{\left[p_{1}^{\prime }p_{2}^{\prime }\to p_{1}p_{2}\right]}$. Therefore, we obtain the relation $\chi_{\left[12\to 1^{\prime }2^{\prime }\right]}=\chi_{\left[1^{\prime }2^{\prime }\to 12\right]}$.

These two collision contributions can be further written down as (denoting $d\varsigma_{i}:=d^{3}r_{i}\;d^{3}p_{i}$)

(42) $$\begin{array}{cc} \Delta^{\left(-\right)} & =\int F\left(p_{1}r_{1};\;p_{2}r_{2},t\right)\chi_{\left[12\to 1^{\prime }2^{\prime }\right]}\;d\varsigma_{1}^{\prime }\;d\varsigma_{2}^{\prime }\;d\varsigma_{2},\\ \Delta^{\left(+\right)} & =\int F\left(p_{1}^{\prime }r_{1}^{\prime };\;p_{2}^{\prime }r_{2}^{\prime },t\right)\chi_{\left[1^{\prime }2^{\prime }\to 12\right]}\;d\varsigma_{1}^{\prime }\;d\varsigma_{2}^{\prime }\;d\varsigma_{2}, \end{array}$$

where $F(p_{1}r_{1};\;p_{2}r_{2},t)$ is the two-particle joint probability, and $\chi_{\left[12\to 1^{\prime }2^{\prime }\right]}$ denotes the transition ratio (or scattering matrix) from the initial state $\left(p_{1}r_{1};\;p_{2}r_{2}\right)$ scattered into the final state $\left(p_{1}^{\prime }r_{1}^{\prime };\;p_{2}^{\prime }r_{2}^{\prime }\right)$. Due to the time-reversal and inversion symmetry of the microscopic scattering process, the transition ratios $\chi_{\left[12\to 1^{\prime }2^{\prime }\right]}$ and $\chi_{\left[1^{\prime }2^{\prime }\to 12\right]}$ equal to each other (see Figure 8). Therefore, the above Equation (41) is further written as [denoting $F_{12}:=F\left(p_{1}r_{1};\;p_{2}r_{2},t\right)$]

(43) $$\partial_{t}f\left(p_{1},r_{1},t\right)+\frac{p_{1}}{m}\cdot \nabla_{r_{1}}f=\int \left(F_{1^{\prime }2^{\prime }}-F_{12}\right)\chi_{\left[12\to 1^{\prime }2^{\prime }\right]}\;d\varsigma_{1}^{\prime }\;d\varsigma_{2}^{\prime }\;d\varsigma_{2}.$$

Now we adopt the “molecular-disorder assumption”, i.e., the two-particle joint PDF can be approximately written as the product of the two single-particle PDF

(44) $$\begin{array}{cc} F(p_{1}r_{1};p_{2}r_{2},t) & ≃f\left(p_{1},r_{1},t\right)\times f\left(p_{2},r_{2},t\right). \end{array}$$

This assumption is usually known as the Stosszahlansatz, or the molecular chaos hypothesis. The word “Stosszahlansatz” was introduced by Ehrenfest in 1912, and its original meaning is “the assumption of collision number” [8,60,66]. Here we adopted the wording from Boltzmann’s paper in 1896 [64,65]. Essentially this is requiring that the correlation between the two particles is negligible. Then the Boltzmann transport equation is obtained as [denoting $f_{i}:=f\left(p_{i},r_{i},t\right)$]

(45) $$\left[\partial_{t}+\frac{p_{1}}{m}\cdot \nabla_{r_{1}}\right]f\left(p_{1},r_{1},t\right)=\int \left(f_{1^{\prime }}f_{2^{\prime }}-f_{1}f_{2}\right)\chi_{\left[12\to 1^{\prime }2^{\prime }\right]}\;d\varsigma_{1}^{\prime }\;d\varsigma_{2}^{\prime }\;d\varsigma_{2}.$$

### 4.2. H-theorem and the Steady State

Now we further review how to prove the *H*-theorem from the above Boltzmann equation, and find out its steady state. Defining the Boltzmann *H*-function as $H\left[f\left(p_{1},r_{1},t\right)\right]:=\int d\varsigma_{1}\;f_{1}\ln f_{1}$, the Boltzmann Equation (45) guarantees H(t) decreases monotonically (dH/dt≤0), and this is the *H*-theorem.

To prove this theorem, we put the Boltzmann equation into the time derivative $\frac{d}{dt}H\left(t\right)=\int d\varsigma_{1}\;\partial_{t}f\left(p_{1},r_{1},t\right)\cdot \ln f\left(p_{1},r_{1},t\right)$. The Liouville diffusion term gives (denoting v:=p/m)

(46) $$\int d\varsigma_{1}\;\frac{p_{1}}{m}\cdot \nabla_{r_{1}}f_{1}\cdot \ln f_{1}=\int d\varsigma \;\nabla_{r}\cdot \left(vf\ln f-vf\right),$$

which can be turned into a surface integral and vanishes. In addition, the collision term gives

(47) $$\begin{array}{cc} \frac{dH}{dt}= & \int \left(f_{1^{\prime }}f_{2^{\prime }}-f_{1}f_{2}\right)\chi_{\left[12\to 1^{\prime }2^{\prime }\right]}\ln f_{1}\;d\varsigma_{1}^{\prime }d\varsigma_{2}^{\prime }d\varsigma_{1}d\varsigma_{2}\\ = & \frac{1}{2}\int \left(f_{1^{\prime }}f_{2^{\prime }}-f_{1}f_{2}\right)\chi_{\left[12\to 1^{\prime }2^{\prime }\right]}\ln f_{1}\;d\varsigma_{1}^{\prime }d\varsigma_{2}^{\prime }d\varsigma_{1}d\varsigma_{2}+\frac{1}{2}\int \left(f_{2^{\prime }}f_{1^{\prime }}-f_{2}f_{1}\right)\chi_{\left[21\to 2^{\prime }1^{\prime }\right]}\ln f_{2}\;d\varsigma_{2}^{\prime }d\varsigma_{1}^{\prime }d\varsigma_{2}d\varsigma_{1}\\ = & \frac{1}{2}\int \left[\left(f_{1^{\prime }}f_{2^{\prime }}-f_{1}f_{2}\right)\chi_{\left[12\to 1^{\prime }2^{\prime }\right]}\ln\left(f_{1}f_{2}\right)\right]d\varsigma_{1}^{\prime }d\varsigma_{2}^{\prime }d\varsigma_{1}d\varsigma_{2}, \end{array}$$

where the second line is because exchanging the integral variables 1↔2 gives the same value. We can further apply the similar trick by exchanging the integral variables $\left(12\right)↔\left(1^{\prime }2^{\prime }\right)$, and that gives

(48) $$\frac{dH}{dt}=\frac{1}{4}\int \left(f_{1^{\prime }}f_{2^{\prime }}-f_{1}f_{2}\right)\left(\ln f_{1}f_{2}-\ln f_{1^{\prime }}f_{2^{\prime }}\right)\chi_{\left[12\to 1^{\prime }2^{\prime }\right]}\;d\varsigma_{1}^{\prime }d\varsigma_{2}^{\prime }d\varsigma_{1}d\varsigma_{2}.$$

Here the transition ratio $\chi_{\left[12\to 1^{\prime }2^{\prime }\right]}$ is non-negative, and notice that $\left(f_{1^{\prime }}f_{2^{\prime }}-f_{1}f_{2}\right)\left(\ln f_{1}f_{2}-\ln f_{1^{\prime }}f_{2^{\prime }}\right)\leq 0$ always holds for any PDF $f_{i}$. Therefore, we obtain dH/dt≤0, which means the function H(t) decreases monotonically, and this encloses the proof.

In the above inequality, the equality holds if and only if $f_{1}f_{2}=f_{1^{\prime }}f_{2^{\prime }}$, which means the collision induced increase $\Delta^{\left(+\right)}$ and decrease $\Delta^{\left(-\right)}$ of f(p,r) must balance each other everywhere, thus it is also known as the detailed balance condition.

The time-independent steady state of f(p,r,t) can be obtained from this detailed balance equation $f_{1}f_{2}=f_{1^{\prime }}f_{2^{\prime }}$. Taking the logarithm of the two sides, it gives

(49) $$\ln f\left(p_{1},r_{1}\right)+\ln f\left(p_{2},r_{2}\right)=\ln f\left(p_{1}^{\prime },r_{1}^{\prime }\right)+\ln f\left(p_{2}^{\prime },r_{2}^{\prime }\right).$$

Notice that the two sides of the above equation depends on different variables, and has a conservation form. Therefore, lnf must be a combination of some conservative quantities. During the collision $\left(p_{1}r_{1};\;p_{2}r_{2}\right)↔\left(p_{1}^{\prime }r_{1}^{\prime };\;p_{2}^{\prime }r_{2}^{\prime }\right)$, the particles collides at the same position, and the total momentum and energy are conserved, thus lnf must be their combinations, namely, $\ln f=C_{0}+C_{1}\cdot p+C_{2}p^{2}$, where $C_{0}$, $C_{1}$, $C_{2}$ are constants. Therefore, f(p,r) must be a Gaussian distribution of p at any position r.

Furthermore, the diffusion term in Equation (45) requires $p\cdot \nabla_{r}f=0$ in the steady state, thus f(p,r) must be homogenous for any position r. The average momentum 〈p〉 should be 0 for a stationary gas. Therefore, the steady state of the Boltzmann Equation (45) is a Gaussian distribution $f\left(p,r\right)∼\exp\left[-p^{2}/2{\bar{p}}_{T}^{2}\right]$ independent of the position r, which is the MB distribution.

### 4.3. Molecular-Disorder Assumption and Loschmidt Paradox

In the above two sections, we demonstrated all the critical steps deriving the Boltzmann equation. Notice that there is no special requirement for the interaction form of the collisions, as long as it is short-ranged so as to make sure only bipartite collisions exist. The contribution of the collision interaction is implicitly contained in the transition rate $\chi_{\left[12\to 1^{\prime }2^{\prime }\right]}$, and the only properties we utilized are (1) $\chi_{\left[12\to 1^{\prime }2^{\prime }\right]}\geq 0$ and (2) $\chi_{\left[12\to 1^{\prime }2^{\prime }\right]}=\chi_{\left[1^{\prime }2^{\prime }\to 12\right]}$. Thus it does not matter whether the interaction is nonlinear.

No doubt to say, the molecular-disorder assumption ($F_{12}≃f_{1}\times f_{2}$) is the most important basis in the above derivations. As mentioned by Boltzmann (pp. 29 in Reference [65]), “*…The only assumption made here is that the velocity distribution is molecular-disordered* (namely, $F_{12}≃f_{1}\times f_{2}$ in our notation) *at the beginning, and remains so. With this assumption, one can prove that H can only decrease, and also that the velocity distribution must approach that of Maxwell*.” Before this approximation, indeed Equation (43) is still formally exact. Clearly, the validity of this assumption, which is imposed on the particle correlations, determines whether the Boltzmann Equation (45) holds. Now we will re-examine this assumption as well as the Loschmidt paradox.

Once two particles collide with each other, they get correlated. In a dilute gas, collisions do not happen very frequently, and once two particles collides with each other, they could hardly meet each other again. Therefore, if initially there is no correlations between particles, we can expect that, on average, the collision induced bipartite correlations are negligibly small, and thus this molecular-disorder assumption holds well.

Now we look at the situation in the Loschmidt paradox. First, the particles experience a “forward” diffusion process for a certain time. According to the *H*-theorem, the entropy increases in this process. Then suppose all the particle momenta are suddenly reversed at this moment. From this initial state, the particles are supposed to evolve “backward” exactly along the incoming trajectory, and thus exhibit an entropy decreasing process, which is contradicted with the *H*-theorem conclusion, and this is the Loschmidt paradox.

However, we should notice that, indeed the first “forward” evolution has established significant (although very small in quantity) bipartite correlations in this new “initial state” [28]. This is quite similar like the above discussion about the momentum-position correlation in Section 3.6. Thus, the above molecular-disorder assumption $F_{12}≃f_{1}\times f_{2}$ does not apply in this case. As a result, the next “backward” evolution is indeed unsuitable to be described by Boltzmann Equation (45). Therefore, the entropy increasing conclusion of the *H*-theorem (dH/dt≤0) does not need to hold for this “backward” process. As well, the preparation of such a specific initial state is definitely unfeasible in practice, thus the “backward” entropy decreasing process is rarely seen.

We emphasize that the Boltzmann Equation (45) is about the single-particle PDF f(p,r,t), which is obtained by averaging over all the other N−1 particles from the full ensemble $\rho (\vec{P},\vec{Q},t)$. Clearly, f(p,r,t) omits much information in $\rho (\vec{P},\vec{Q},t)$, but indeed it is enough to give most macroscopic thermodynamic quantities. For example, the average kinetic energy of each single molecular $\langle p^{2}\rangle =\int d\varsigma \;p^{2}f\left(p,r\right)$ determines the gas temperature *T*, and the gas pressure on the wall is given by $P=\int_{p_{x}>0}d\varsigma \;\left(2p_{x}\right)\cdot v_{x}f\left(p,r\right)$ [1].

In contrast, indeed the inter-particle correlations ignored by the single-particle PDF f(p,r,t) are quite difficult to be sensed in practice. Therefore, the *N*-particle ensemble may be “inferred” as ${\tilde{\rho }}_{\inf}\left(\vec{P},\vec{Q},t\right)=\prod_{i=1}^{N}f\left(p_{i},r_{i},t\right)$, which clearly omits the inter-particle correlations in the exact $\rho (\vec{P},\vec{Q},t)$. Similar like the discussion in Section 3.4, based on this inferred ensemble ${\tilde{\rho }}_{\inf}\left(\vec{P},\vec{Q},t\right)$, the entropy change gives $\Delta_{i}S=S_{G}\left[{\tilde{\rho }}_{\inf}\left(t\right)\right]-S_{G}\left[{\tilde{\rho }}_{\inf}\left(0\right)\right]=N\ln\left(V/V_{0}\right)$, which exactly reproduces the result in standard thermodynamics. Thus $\Delta_{i}S$ indeed characterizes the increase of the inter-particle correlations.

In sum, even in the presence of the particle collisions, the full *N*-particle ensemble $\rho (\vec{P},\vec{Q},t)$ still follows the Liouville equation exactly, thus its Gibbs entropy does not change. On the other hand, the single-particle PDF f(p,r,t) follows the Boltzmann equation, thus its entropy keeps increasing until reaching the steady state. In addition, this roots from our ignorance of the inter-particle correlations in the full $\rho (\vec{P},\vec{Q},t)$.

## 5. Summary

In this paper, we study the correlation production in open and isolated thermodynamic systems. In a many-body system, the microscopic dynamics of the whole system obeys the time-reversal symmetry, which guarantees the entropy of the global state does not change with time. Based on the microscopic dynamics, indeed the full ensemble state is not evolving towards the new equilibrium state as expected from the macroscopic intuition. However, the correlation between different local DoF, as measured by their mutual information, generally increases monotonically, and its amount could well reproduce the entropy increase result in the standard macroscopic thermodynamics.

In open systems, as described by the second law in standard thermodynamics, the irreversible entropy production increases monotonically. It turns out that this irreversible entropy production is just equal to the correlation production between the system and its environment. Thus, the second law can be equivalently understood as the system-bath correlation is increasing monotonically, while at the same time, the s + b system as a whole still keeps constant entropy.

In isolated systems, there is no specific partition for “system” and “environment”, but we could see the momentum and spatial distributions, as the marginal distributions of the total ensemble, exhibit the macroscopic irreversibility, and their correlation increases monotonically, which reproduces the entropy increase result in standard thermodynamics. In the presence of particle collisions, different particles are also establishing correlations between each other. As a result, the single-particle distribution exhibits the macroscopic irreversibility as well as the entropy increase in standard thermodynamics, which is just the result of the Boltzmann *H*-theorem. At the same time, the full ensemble $\rho (\vec{P},\vec{Q},t)$ of the many-body system still follows the Liouville equation, which guarantees its entropy does not change with time.

It is worth noticing that, in practice, usually it is the partial information (e.g., marginal distribution, few-body observable expectations) that is directly accessible to our observation. However, indeed most macroscopic thermodynamic quantities are obtained only from such partial information like the one-body distribution, and that is why they exhibits irreversible behaviors. However, due to the practical restrictions in measurements, the dynamics of the full ensemble state, such as the constant entropy behavior, is quite difficult to be sensed in practice.

In sum, the global state keeps constant entropy, while partial information exhibits the irreversible entropy increase. However, in practice, it is the partial information that is directly observed. In this sense, the macroscopic irreversible entropy increase does not contradict with the microscopic reversibility. Clearly, such correlation production understanding can be applied for both quantum and classical systems, no matter whether there exist complicated particle interactions, and it can be well used for time-dependent non-equilibrium states. Moreover, it is worths noticing that, if the bath of an open system is a non-thermal state, indeed this is beyond the application scope of the standard thermodynamics, but we could see such correlation production understanding still applies in this case. We notice that such correlations can be found in many of recent studies of thermodynamics, and it is also quite interesting to notice that similar idea can be used to understand the paradox of blackhole information loss, where the mutual information of the radiation particles is carefully considered [67,68,69,70].

## Appendix A. The Entropy of a Continuous Probability Distribution

For a finite sample space of *N* events with probabilities $\left\{p_{n}\right\}$, the information entropy is well-defined as $S\left[\{p_{n}\}\right]:=-\sum_{n}p_{n}\ln p_{n}$. However, the situation of a continuous PDF is not so trivial, and a simple generalization from the discrete case would lead to a divergency problem.

For example, considering a uniform PDF P(x)=1/L in the area x∈[0,L], to calculate its entropy, we first divide the area into *N* pieces averagely, and then turn the discrete summation into the continuous integral by taking the limit N→∞. Clearly here each piece takes the probability $p_{n}=1/N$, thus the entropy is $S=-\sum_{n}\frac{1}{N}\ln\frac{1}{N}=\ln N$, but it diverges when N→∞.

Generally, for a continuous PDF P(x) in the area x∈[0,L], after the division into *N* pieces, the entropy is

(A1) $$S^{\left(N\right)}=-\sum_{n=0}^{N-1}P\left(x_{n}\right)\Delta x\cdot \ln\left[P(x_{n})\Delta x\right],$$

where Δx=L/N, and $x_{n}=n\cdot \Delta x$. Remember here P(x) is the probability density, which has the unit of the length inverse $\left[L^{-1}\right]$, and P(x)Δx is the unitless probability. However, when taking the limit N→∞, the above equation becomes

(51) $$\begin{array}{cc} \lim_{N\to \infty }S^{\left(N\right)} & =\lim_{N\to \infty }\sum_{n=0}^{N-1}-P\left(x_{n}\right)\ln P\left(x_{n}\right)\cdot \Delta x-P\left(x_{n}\right)\cdot \Delta x\ln\frac{L}{N}\\ & =-\int_{0}^{L}dx\;P\left(x\right)\ln P\left(x\right)-\int_{0}^{L}dx\;P\left(x\right)\ln L+\lim_{N\to \infty }\ln N\cdot \sum_{n=0}^{N-1}P\left(x_{n}\right)\Delta x\\ & :=-\int_{0}^{L}dx\;P\left(x\right)\ln\left[P\left(x\right)\cdot L\right]+\ln N. \end{array}$$

In the 3rd term of the 2nd line, the summation converges to 1 when N→∞, thus this term diverges as ∼lnN, and we denote it as lnN. In addition, notice that in the 1st term, now [P(x)·L] appears in the logarithm as a whole unitless quantity.

The diverging term lnN cannot be simply omitted. Considering the above example of the uniform distribution P(x)=1/L in the area x∈[0,L], which is supposed to give the largest entropy, we can see the 1st term in the above result gives 0, and its entropy is exactly given by this diverging term $S^{\left(N\right)}=\ln N$.

Therefore, when generalizing the information entropy for the continuous PDF, it always contains a diverging term lnN. There are two ways to resolve this problem. First, whenever this entropy is under discussion, we always focus on the entropy difference between two states but not their absolute values. For example, in Section 3.4 we always focus on the entropy change ΔS:=S(t)−S(0) comparing with the initial state. In this case, the divergency of lnN in S(t) and S(0) just cancels each other. As well, the length *L* in the above ln[P(x)·L] also can be canceled. Therefore, in the definition (39) for $S_{x}$ and $S_{p}$, the probability densities $P_{x}\left(x\right)$ and $P_{p}\left(p\right)$ appear in the logarithm directly although they are not unitless.

Second, from the above simple example of the uniform PDF, we can see indeed this divergency origins from the idealization for the continuity of the probability distribution. Notice that a continuous probability density is indeed not directly accessible in practical measurements, in contrast, we should first divide the continuous area x∈[0,L] into *N* intervals, and then measure the probability that appears in the interval between $x_{n}$ and $x_{n}+\Delta x$, which is denoted as $p_{n}=P\left(x_{n}\right)\Delta x$. In the limit Δx→0, the histogram $P(x_{n})$ becomes the continuous probability density (see Section 3.5). Indeed here Δx is the resolution in the measurement. A finer resolution indicates a larger sample space and more possible probability distributions, and that results to the divergence of lnN. However, most practical measurements have certain resolution limit, thus Δx cannot approach 0 infinitely. If this resolution restriction is considered, the diverging term lnN is constrained by the finite resolution.
